# Comparative analysis of the predicted secretomes of Rosaceae scab pathogens *Venturia inaequalis* and *V. pirina* reveals expanded effector families and putative determinants of host range

**DOI:** 10.1186/s12864-017-3699-1

**Published:** 2017-05-02

**Authors:** Cecilia H. Deng, Kim M. Plummer, Darcy A. B. Jones, Carl H. Mesarich, Jason Shiller, Adam P. Taranto, Andrew J. Robinson, Patrick Kastner, Nathan E. Hall, Matthew D. Templeton, Joanna K. Bowen

**Affiliations:** 1 The New Zealand Institute for Plant & Food Research Limited (PFR), Auckland, New Zealand; 2 Animal, Plant & Soil Sciences Department, AgriBio Centre for AgriBioscience, La Trobe University, Melbourne, Victoria Australia; 3 Plant Biosecurity Cooperative Research Centre, Bruce, ACT Australia; 4 The School of Biological Sciences, University of Auckland, Auckland, New Zealand; 5 Plant Sciences Division, Research School of Biology, The Australian National University, Canberra, Australia; 6 Life Sciences Computation Centre, Victorian Life Sciences Computation Initiative (VLSCI), Victoria, Australia; 7 Present Address: The Centre for Crop and Disease Management, Curtin University, Bentley, Australia; 8 Present Address: Institute of Agriculture & Environment, Massey University, Palmerston North, New Zealand; 9 Present Address: INRA-Angers, Beaucouzé, Cedex, France

**Keywords:** *Venturia inaequalis*, *Venturia pirina*, Apple, *Malus* x *domestica*, European pear, *Pyrus communis*, Secretome, Effector

## Abstract

**Background:**

Fungal plant pathogens belonging to the genus *Venturia* cause damaging scab diseases of members of the Rosaceae. In terms of economic impact, the most important of these are *V. inaequalis*, which infects apple, and *V. pirina*, which is a pathogen of European pear. Given that *Venturia* fungi colonise the sub-cuticular space without penetrating plant cells, it is assumed that effectors that contribute to virulence and determination of host range will be secreted into this plant-pathogen interface. Thus the predicted secretomes of a range of isolates of *Venturia* with distinct host-ranges were interrogated to reveal putative proteins involved in virulence and pathogenicity.

**Results:**

Genomes of *Venturia pirina* (one European pear scab isolate) and *Venturia inaequalis* (three apple scab, and one loquat scab, isolates) were sequenced and the predicted secretomes of each isolate identified. RNA-Seq was conducted on the apple-specific *V. inaequalis* isolate Vi1 (in vitro and infected apple leaves) to highlight virulence and pathogenicity components of the secretome. Genes encoding over 600 small secreted proteins (candidate effectors) were identified, most of which are novel to *Venturia*, with expansion of putative effector families a feature of the genus. Numerous genes with similarity to *Leptosphaeria maculans AvrLm6* and the *Verticillium* spp. *Ave1* were identified. Candidates for avirulence effectors with cognate resistance genes involved in race-cultivar specificity were identified, as were putative proteins involved in host-species determination. Candidate effectors were found, on average, to be in regions of relatively low gene-density and in closer proximity to repeats (e.g. transposable elements), compared with core eukaryotic genes.

**Conclusions:**

Comparative secretomics has revealed candidate effectors from *Venturia* fungal plant pathogens that attack pome fruit. Effectors that are putative determinants of host range were identified; both those that may be involved in race-cultivar and host-species specificity. Since many of the effector candidates are in close proximity to repetitive sequences this may point to a possible mechanism for the effector gene family expansion observed and a route to diversification via transposition and repeat-induced point mutation.

**Electronic supplementary material:**

The online version of this article (doi:10.1186/s12864-017-3699-1) contains supplementary material, which is available to authorized users.

## Background

Plant-pathogen interactions are a fine interplay between prospective host and pathogen, involving the exchange of molecular signals that determine the outcome of the interaction. The plant endeavours to detect invaders by deploying, as a first line of defence, pattern recognition receptors that recognise pathogen-associated molecular patterns (PAMPs) shared by a wide range of non-specialised microbes, with a resulting induction of a low-level defence response termed PAMP-triggered immunity (PTI). In response, pathogens evolve effectors that enhance a pathogen’s ability to cause disease, often by blocking or suppressing PTI [1–4]. Plants in turn evolve resistance (R) proteins that recognise a subset of these effectors either directly or by their actions on plant host targets. Thus the respective effector and R protein complements can contribute to the determination of host-range for any given pathogen.

The genus *Venturia* belongs to the order Venturiales, which is assigned to the class Dothideomycetes [5]. The Dothideomycetes include many highly destructive plant pathogens, the *Venturiales* being no exception. *Venturia* pathogens are relatively host-specific and infect selected members of the family Rosaceae, perhaps the best known and most widely researched of which is *V. inaequalis* Cooke (Wint.) that causes the economically important disease apple scab [6, 7]. Related species, for example *V. pirina*, *V. nashicola*, *V. carpophila* and *V. cerasi*, cause scab diseases of other Rosaceae hosts: European pear, Asian pear, peach and cherry respectively [8–11]. *Venturia* species cause similar symptoms by adopting analogous biotrophic parasitic strategies [8–10]. During infection they penetrate the host cuticle directly and then develop stromata (laterally dividing, pseudoparenchymatous cells) in the cuticle and sub-cuticular space without penetrating host cells. Ultimately conidiophores and conidia differentiate from stromata and erupt through the cuticle resulting in the formation of dark, crusty, dry lesions (scabs) on leaves and fruit. A fundamental question arises as to what determines the host range of these pathogens, especially given that they have similar modes of biotrophic parasitism and infect closely related host species.

A further level of complexity underlies pathogen/host species specificity in scab fungi, for example, certain isolates classified as *V. inaequalis* on the basis of morphological and molecular criteria, as well as their ability to mate, are unable to infect *Malus*, but instead infect different Rosaceous hosts, such as *Eriobotrya* (loquat) and *Pyracantha* (firethorn) [12, 13]. Whether these isolates should be considered as separate species or formae speciales is still open to debate. Gladieux and associates [13] suggested that there should be no sub-species delineation based on analysis of six nuclear loci. These isolates are therefore very closely related phylogenetically, but have distinct host specificities. Host cultivar specificity has long been demonstrated in isolates of *V. inaequalis* that infect *Malus* [14]; 17 gene-for-gene pairings have been identified to date between races of *V. inaequalis* and cultivars of *Malus*. Only two scab *R* gene loci in apple (*Rvi6* and *Rvi15*) have been fully characterised [15–18]; however, no effectors of *V. inaequalis* have been characterised to date*.* Work to identify the *V. inaequalis* effector repertoire that determines cultivar specificity, and most probably host specificity, has been impeded by the lack of a whole genome sequence for this species.

Genome estimates for *V. inaequalis* range from 38 Mb [19] to 100 Mb [20]. The de novo transcriptome of a single isolate of *V. inaequalis* has been published [21], with analysis of both in vitro and in planta transcripts. In our study the whole genome sequences of four isolates of *V. inaequalis* (three physiological races, differing in their ability to infect apple accessions carrying different *R* genes, and a loquat-infecting isolate) were sequenced using Illumina sequencing technologies. The *V. inaequalis* genomes were compared to a single *V. pirina* genome [22]. Given the extracellular in planta niche the *Venturia* fungi occupy during parasitism it can be assumed that the majority of the factors that enable successful parasitism are first secreted into the sub-cuticular plant-pathogen interface. Therefore the secretomes of these isolates are of utmost relevance and so were compared to identify putative pathogencity or virulence factors that may have a role in host determination, with special emphasis on secreted enzymes and putative proteinaceous effectors.

## Results

### Whole genome assemblies and gene predictions

A total of 40 Mb (Vi1.2.8.9) to 61 Mb (Vi1.10) was assembled for *Malus*-infecting *V. inaequalis* isolates. The assembled genome size for the loquat scab pathogen (ViL) was 62 and 41 Mb for the pear scab pathogen (Vp). Lack of sequencing of mate-pair libraries with long inserts contributed to the Vi1.2.8.9 assembly having the lowest N50 value (~49 kb) among the five isolates, while Vp had the largest N50 (332 kb, Table 1, Additional file 1). A summary of the repeat content masked for each genome assembly is presented in Table 1 and Additional file 2.

**Table 1:** The whole genome assemblies of isolates of *Venturia inaequalis* and *V. pirina*

Species	*Venturia inaequalis*				*Venturia pirina*
Isolate (race)	Vi1 (race 1) = ICMP13258 = MNH120	Vi1.10 (race 1,10) = EU-B04^d^	Vi1.2.8.9 (race 1,2,8,9) = 1639	ViL = 1389	Vp = ICMP11032
Host range	*Malus* × *domestica*	*Malus* × *domestica*	*Malus* × *domestica*	*Eriobotrya japonica*	*Pyrus communis*
Estimated genome coverage	120x	90x	89x	89x	74x
Package used for assembly	Velvet	ALLPATHS -LG	Velvet	ALLPATHS -LG	Velvet
Number of scaffolds	1012	1415	1680	1040	364
Size (Mb)	55	61	40	62	41
N50	233760	136376	48770	213378	332167
N90	16289	23736	11843	37088	81548
Repeats in total genome (%)	4.11%	33.41%	0.74%	35.60%	7.28%
Number of predicted genes^a^	13333/12546	12234	12868	12258	11960
Percentage completeness: partial (full)^b^	99.60 (99.19)	98.79 (95.16)	98.39 (94.76)	97.58 (94.76)	98.39 (95.16)
Percentage completeness: partial (full)^c^	99.51 (96.45)	99.37 (97.01)	99.37 (96.94)	99.51 (97.22)	99.65 (97.01)

^a^Hybrid with hints/ab initio gene predictions for Vi1

^b^CEGMA analysis using both partial and full gene sequences [25]

^c^BUSCO analysis using both partial and full gene sequences [26]

^d^Note the recent race designation change reported for EU-B04 [187], previously reported as race (1,14) by Bus et al. [14]

Gene models were predicted with AUGUSTUS gene prediction software (Table 1 and Additional file 3) [23, 24]. The number of gene models in the *V. inaequalis* isolates ranged from 12,234 to 13,333 whereas there were 11,960 identified in *V. pirina*. To estimate the completeness of the assembled genomes, the assemblies were scanned for the 248 most highly conserved core eukaryotic genes using the Core Eukaryotic Genes Mapping Approach (CEGMA; Table 1 and Additional file 4) [25]. Comparisons of partial or complete predicted translated proteins demonstrated that the *V. inaequalis* Vi1 genome is the most complete, with only one or two core eukaryotic genes missing respectively. The lowest representation of the 248 core eukaryotic genes in any genome was still high, 235 complete and another seven partial predicted proteins for *V. inaequalis* ViL, indicating a reasonably complete whole genome sequence for all isolates. In addition, a Benchmarking Universal Single-Copy Orthologs (BUSCO) analysis was also conducted using BUSCO_v1.1b [26]. This analysis indicated that the *V. pirina* genome is the most complete and of the *V. inaequalis* genomes Vi1 and ViL are the most complete when partial predicted proteins are considered, although on comparison of complete predicted proteins the ViL genome is the most complete (Table 1 and Additional file 5). Since Vi1 was the most comprehensive *V. inaequalis* genome in terms of coverage when CEGMA and BUSCO analyses were taken together, and is predicted to have the most complete set of avirulence effectors (due to the avirulent phenotype of this isolate on all but one of the set of resistant apple host differentials following inoculation in glasshouse trials; Vincent Bus, personal communication), this isolate was selected for preliminary expression (RNA-Seq) analyses.

### The predicted secretomes of *Venturia inaequalis* and *V. pirina*

**Fig. 1:**
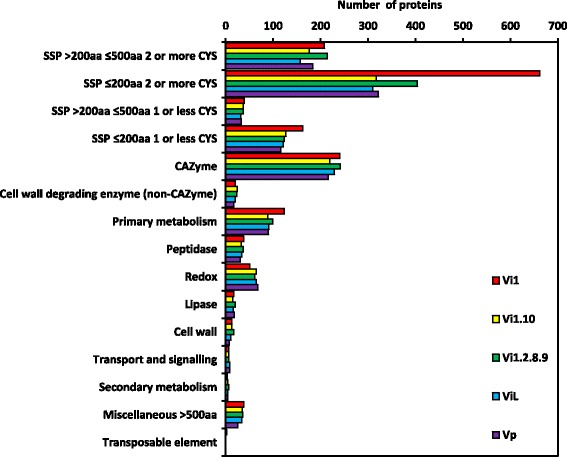
The secretomes of four isolates of *V. inaequalis* and one isolate of *V. pirina*. Annotations were based on gene ontology analysis including interrogation of NCBI RefSeq [168], InterPro [169], UniRef [170], ExPASy UniProtKB/Swiss-Prot [171] and ExPASy Prosite [172], CAZyme identification using the CAT server [27] and BLASTp searches against the NCBI non-redundant database [29]. Small secreted proteins (SSPs) include predicted proteins with similarity to known effectors and proteins with no known function, either with or without putative conserved motifs identified by PfamScan [36–38]

To predict the secretome of each isolate, gene models were analysed with a pipeline of programmes (Additional file 6). The total number of genes encoding the predicted secretome for each genome is as follows: 1622 in Vi1; 1158 in Vi1.10; 1324 in Vi1.2.8.9; 1131 in ViL and 1139 in Vp. The number of predicted proteins in the Vi1 secretome is greater than that for the other genomes due to use of both ab initio (based on the trained species model) and hybrid (evidence informed) gene predictions in the analysis. Thakur et al. [21] reported a lower predicted secretome number of 946, however this was based on transcriptome data alone and, as such, direct comparison is problematic. Gene ontology analysis through an in-house annotation pipeline (BioView; Ross Crowhurst, Marcus Davy and Cecilia Deng, unpublished) was used to annotate predicted genes in each secretome; however, the majority of the predicted proteins had no gene ontology classification. Hence, for this reason, the Carbohydrate Active Enzyme (CAZyme) Analysis Toolkit (CAT) [27], and similarity searches utilising BLASTp [28] against the NCBI non-redundant (nr) database [29] were used in addition to gene ontology. Where conflicting designations occurred, CAT, then gene ontology took precedence over similarity search designations. The secretome repertoire, in terms of diversity and abundance of protein classes, was similar between all *Venturia* isolates, with small, secreted, non-enzymatic proteins (SSPs) dominating (Fig. 1). For comparative purposes the secretomes of three plant pathogenic fungi, *Parastagonospora nodorum* (synonym: *Stagonospora nodorum*), *Cladosporium fulvum* and *Puccinia graminis* f. sp *tritici* were also identified using the same pipeline of programmes using predicted proteins downloaded from the Joint Genome Institute (JGI; Additional files 6 and 7). The size of the *P. graminis* f. sp *tritici* secretome (1820 predicted proteins) was significantly larger than that recorded for *C. fulvum* and *P. nodorum* (1170 and 1122 predicted proteins respectively). The size of the Vi1 secretome was similar to that of *P. graminis* f. sp. *tritici*, whereas the remaining *Venturia* secretomes were of a similar size to those of *P. nodorum* and *C. fulvum*.

**Fig. 2:**
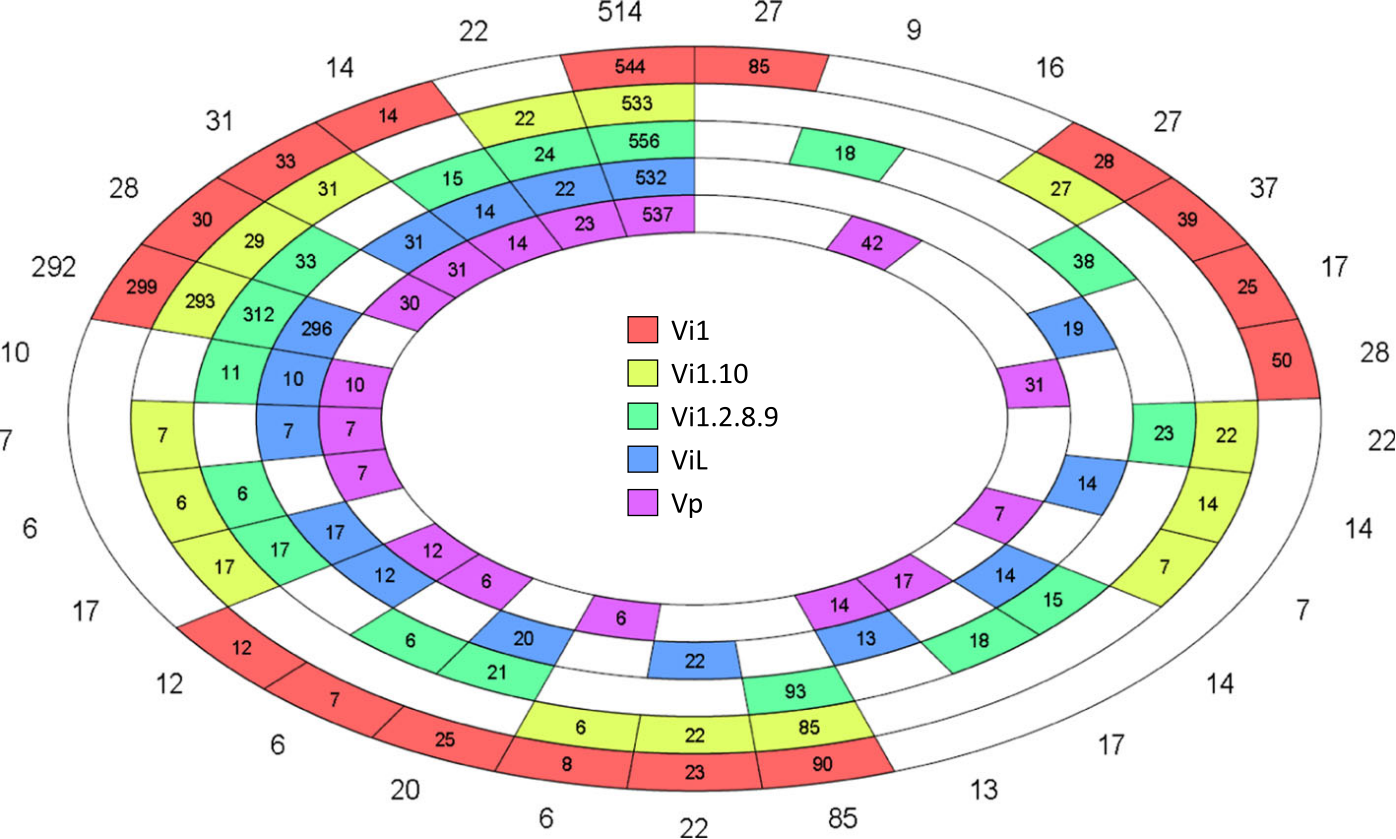
Proteins in the secretomes of four isolates of *Venturia inaequalis* and one of *V. pirina*. Similar proteins in each of the secretomes were identified by OrthoMCL-2.0.3 [30] together with the Markov clustering algorithm mcl-09-149 [173]. Similarity levels were calculated based on reciprocal BLASTp similarity searches between the protein sequences with an e value threshold of 1e-10. Figures within the boxes represent the number of proteins in each cluster, whereas figures outside the wheel are the number of clusters in each sector. Those proteins that are singletons within each secretome are not represented

OrthoMCL was conducted to assess similarity between the predicted *Venturia* secretomes (Fig. 2 and Additional file 8) [30]. A total of 898 isolate-specific singletons were identified (310 in Vi1; 37 in Vi1.10; 118 in Vi1.2.8.9; 88 in ViL, and 345 in Vp) but are not included in the OrthoMCL clustering figure (Fig. 2). The *Venturia* core secretome (those predicted secreted proteins represented in all *Venturia* isolates) consisted of 514 orthologous clusters, with CAZymes (163) and non-enzymatic SSPs (225) being the predominant classes (Additional file 8). The *Venturia* pan secretome (the sum of all predicted secreted proteins from all *Venturia* isolates) totalled 5474 representing 2238 individual proteins (1340 clusters, plus 898 isolate-specific singletons), with many lineage-specific and host-specificity candidates identified, most of which were predicted to encode non-enzymatic SSPs. The majority of these SSPs had no significant similarity to proteins in the public domain. The *V. inaequalis*-unique (including both apple- and loquat-infecting strains) pan secretome totalled 2033 representing 1156 individual proteins (603 clusters and 553 singletons), with a total of 929 representing 672 individual proteins (207 clusters plus 465 singletons) specific to apple-infecting isolates, with 85 of the clusters (mostly with a single protein from each isolate) found in all three isolates. There were 88 singletons, but no unique clusters specific to the loquat-infecting isolate ViL. The *V. pirina*-specific secretome totalled 480 representing 387 individual proteins, with 16 unique protein clusters and 345 singletons.

**Fig. 3:**
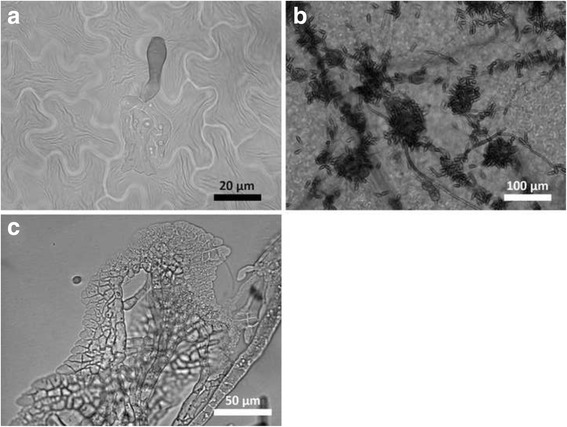
Microscopic evaluation of *Venturia inaequalis* Vi1 infection of susceptible apple leaves. **a** Two, and **b** seven days post inoculation, and **c** in vitro (in cellophane) showing stromatic growth habit

**Fig. 4:**
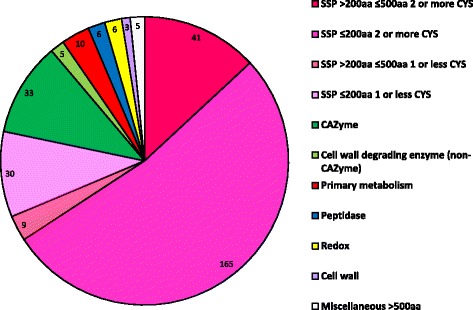
Predicted proteins present in the *V*IS set from the secretome of *Venturia inaequalis* isolate Vi1. Annotations were based on gene ontology analysis including interrogation of NCBI RefSeq [168], InterPro [169], UniRef [170], ExPASy UniProtKB/Swiss-Prot [171] and ExPASy Prosite [172], CAZyme identification using the CAT server [27] and BLASTp searches against the NCBI non-redundant database [29]. Small secreted proteins (SSPs) include predicted proteins with similarity to known effectors and proteins with no known function, either with or without putative conserved motifs identified by PfamScan [36–38]. The order of the categories in the legend is the same as that in the chart

Two sets of RNA-Seq data, from libraries made with RNA harvested from *V. inaequalis* Vi1 grown in vitro (in cellophane membranes) and Vi1-infected apple seedling leaves (two and seven days post inoculation; dpi), provided evidence of transcription for 93.4% of the Vi1 secretome. The RNA-Seq data were also used to select a smaller subgroup of the Vi1 secretome to analyse in greater detail (sequences are deposited under the BioProject ID PRJNA261633: in vitro: SRR1586226, two dpi in planta: SRR1586224, seven dpi in planta: SRR15862230). These data were used as indicators of involvement in virulence or pathogenicity. Figure 3 shows representative stages of this material (no signs of microbial contamination were evident in the sterile distilled water (SDW)-inoculated apple leaves, as assessed by microscopic evaluation). Two approaches were used: first, overall Vi1 gene expression levels were estimated by analysing fragments per kilobase of exon per million reads mapped (FPKM) values. This enabled a ranking of the genes in terms of overall expression during infection to be calculated at both two and seven dpi and the most highly expressed genes at either time point were selected to give the top 5% data set. Of the 82 genes identified, 48 were expressed at two and seven dpi, 17 at two only and 17 at seven dpi only. In addition, a differential expression analysis detected 268 of the 1622 genes that were up-regulated during growth in planta, compared with in vitro growth (log_2_ fold change not smaller than two with a false discovery rate of less than 0.05). Thirty-seven genes were in both the top 5% data set and up-regulated during infection gene sets, giving a total of 313 individual genes (which will be referred to henceforth as the ***V*** *enturia* **I**nfection **S**ecretome or ‘*V*IS’ gene set; Fig. 4).

#### Carbohydrate-Active Enzymes (CAZymes) and putative non-CAZyme cell wall degrading enzymes

The CAZyme repertoire (216 to 240 proteins, depending on the isolate) was similar between all *Venturia* secretomes, with 167 belonging to the core secretome (Additional files 8, 9 and 10). Of these, all isolates have between 16 and 25 proteins with multiple CAZyme domains.

Proteins were identified as being associated with carbohydrate-binding motif (CBM), carbohydrate esterase (CE), glycoside hydrolase (GH), polysaccharide lyase (PL) and glycosyl transferase (GT) domains. Of these, the classes with the greatest number of protein members across each genome were those with: CBM2, CBM1 and CBM13 domains, with specificity for cell wall carbohydrates, and in terms of the latter two, plant cell wall specifically; CE5 domains with specificity for cutin; GH28 with specificity for plant cell wall pectin; GH3 and GH5 with specificity for β-glycans; and GH43 with specificity for pectin and hemicelluloses. Also identified, although not as numerous, were CAZyme domain-containing proteins predicted to interact with fungal cell wall components including: GHs associated with β-glycans, α-glucan, chitin, and CBM domains specific for chitin [31, 32].

The majority of the CAZymes were either present in OrthoMCL clusters that had a single enzyme from each isolate and were thus members of the core secretome set, or in clusters comprised of enzymes from only the four isolates of *V. inaequalis*, not *V. pirina* (Additional files 7 and 9).

Similar numbers of predicted proteins (17 to 24) were identified in the secretome sets, which were similar to enzymes that degrade non-carbohydrate cell-wall components, e.g. ligninases (Fig. 1 and Additional file 8).

#### Peptidases and lipases

Depending on the isolate, 31 to 38 predicted proteins were identified in the *Venturia* secretomes with similarity to peptidases (Figs. 1 and 4, Additional files 2 and 11). The majority of these were conserved in the core secretome or in isolates of *V. inaequalis* rather than *V. pirina*. There were proteins predicted in the secretomes of only the *V. inaequalis* isolates that were similar to AVR-Pita, the avirulence protein from *Magnaporthe* with zinc metalloprotease features that binds directly to its cognate R protein [33, 34], albeit with a relatively high similarity score of 8e-16 recorded when the Pathogen Host Interactions database (PHI-base) [35] was interrogated. In addition, a further similar protein was also encoded by the genome of *V. pirina* but was not predicted to be secreted. There were also between eight and 11 proteins, depending on secretome, with similarity to proteases from *Candida* and *Coccidioides* following interrogation of protein sequences against PHI-base. Predicted proteins with similarity to lipases were also identified in each secretome (15 to 20, depending on the secretome).

#### Primary metabolism and putative proteins involved in redox reactions

Proteins with a wide range of functionality in primary metabolism, e.g. proteins involved in amino acid (aa) or nucleotide metabolism; phosphatases and nitrilases, and unclassified enzymes with an alpha/beta hydrolase fold were identified. Depending on the isolate, there were between 88 and 123 proteins in this category in each secretome, most of which were in the core *Venturia* secretome. Numerous (51 to 68) proteins with putative functions in redox reactions were identified in the *Venturia* secretomes.

#### Putative proteins associated with the cell wall, transport, signalling and secondary metabolism

As expected there were few proteins in the secretomes associated with the cell wall, transport, signalling and secondary metabolism, since these proteins would be eliminated in the secretome prediction pipeline (Additional file 6). The majority of these proteins were members of the core secretome set (Additional file 8).

#### Small secreted proteins

Proteins classified as SSPs (i.e. mature predicted protein ≤500 aa in length, including proteins with similarity to known effectors, but excluding proteins with similarity to lytic enzymes) made up between 55 and 66% of each secretome (Fig. 1 and Additional file 8). For comparative purposes, three secretomes derived from publicly available genomes (housed at the JGI) were also screened for SSPs using the same pipeline of programmes as those used to analyse the *Venturia* secretomes. SSPs (less than 500 aa in length) made up 79% of the secretome of *P. graminis* f. sp. *tritici*, and 43 and 28% of the secretomes of *P. nodorum* and *C. fulvum* respectively. Smaller SSPs less than 200 aa in length predominated in the *Venturia* and *P. graminis* f. sp. *tritici* secretomes, especially those with a predicted signal peptide. In contrast, SSPs of less than 200 aa in length in the predicted SSP repertoires of *C. fulvum* and *P. nodorum* were dominated by those predicted to be secreted by a non-classical mechanism (Additional file 7). The majority of genes encoding SSPs in Vi1 were supported with evidence of transcription (95%). Most SSPs are novel to *Venturia* (i.e. had BLASTp similarity of ≥1e-10 to any non-*Venturia* proteins in public databases) and most of these novel SSPs were ≤200 aa in length. The vast majority of *V*IS genes, 245/313, were predicted to encode SSPs (≤500 aa in length), 195 of which were ≤200 aa in length (Fig. 4). Of these smaller SSPs, 165 have two or more cysteines in the mature predicted protein. Larger secreted proteins (>500 aa in length) with no similarity to known proteins were also represented in each of the secretomes (25 to 38 putative proteins; Fig. 1).

### Candidate effectors from the SSP set

The secretomes of *Venturia* have several proteins with a putative role in binding hydrophobic surfaces including between 12 and 15 proteins with hydrophobic surface binding (HsbA) domains (Pfam: PF12296.3) as identified by PfamScan [36–38], with similarity to the *Aspergillus oryzae* HsbA, Hydrophobic Surface Binding, cell wall galactomannoprotein. Five putative *HsbA-*like genes were identified amongst the Vi1 *V*IS gene set. Another gene up-regulated at seven dpi encodes a small predicted mature protein of 120 aa with four cysteines and shares predicted aa sequence identity (e value: 1e-17) to bacterial chaplins, which are membrane-associated proteins known to have similar functions to hydrophobins [39]. Predicted proteins with similarity to bacterial chaplins are also present in Vi1.10, ViL and Vp, but not Vi1.2.8.9.

Two or three proteins with similarity to fungal hydrophobins were identified in the secretomes of *V. inaequalis* isolates and four in the *V. pirina* secretome, on the basis of conserved cysteine patterns [40, 41]. None were included in the *V*IS gene set.

The *Venturia* secretomes included predicted SSPs with similarity to known effectors from other fungi, including Ecp6 (GenBank: ACF19427.1) from *C. fulvum* [42], AvrLm6 (CAJ90695.1) from *Leptosphaeria maculans* [43] and Ave1 (AFB18185.1) from *Verticillium* spp. [44]. The *Venturia* candidate effectors were most similar to these effectors in a reciprocal BLAST of the *Cladosporium*, *Leptosphaeria* and *Verticillium* genomes. In addition to the OrthoMCL clustering analysis, further similarity searches within genomes were employed with a more relaxed threshold of 1e-5, to ascertain whether putative paralogues (multigene families) were present. Single orthologues of *Ecp6* were identified in each of the *Venturia* genomes analysed, corroborating the data of Thakur et al. [21] who reported an orthologue of *Ecp6* in the transcriptome of a single apple-specific isolate of *V. inaequalis*. A search of the Pfam database [36] with the Vi1 orthologue of Ecp6 revealed three peptidoglycan-binding, lysin motif (LysM) domains. At the aa level there is 42% identity between Ecp6 and the *V. inaequalis* orthologues, whereas the *V. pirina* orthologue has slightly higher identity at 44%. The Vi1 and ViL predicted proteins are identical, whereas that from Vi1.2.8.9 has one aa substitution compared with the Vi1/ViL sequence and that from Vi1.10 has four aa substitutions. The Vi1 *Ecp6* orthologue was expressed during growth in planta and in vitro, although expression was not sufficiently high to be in the *V*IS set.

Orthologues of *AvrLm6* [45] and *Ave1* are present as large gene families in all of the *Venturia* genomes examined. The copy number of full length *AvrLm6*-like genes varied among *V. inaequalis* isolates (24–29 copies), while the *V. pirina* isolate had 16 copies. Further characterisation of the *Venturia AvrLm6*-like gene families is reported in Shiller et al. [45]. Three *AvrLm6*-like genes are present in the *V*IS gene set.

Ten full-length *Ave1-*like genes and seven *Ave1-*like pseudogenes, were identified in the whole genome sequence of *V. inaequalis* Vi1, with similar numbers in all other *Venturia* genomes. The *V. inaequalis* Ave1-like full length, predicted proteins range in similarity from 37 to 57% identity to *V. dahliae* Ave1. All but one of the Vi1 Ave1-like proteins have transcript evidence, with expression at all growth stages, including in vitro (on cellophane), with some very highly expressed during both infection time points. Two *Ave1*-like gene loci are represented in the *V*IS gene set.

Loci of all 16 of the previously identified ***V*** *.* ***i*** *naequalis* **c**andidate **e**ffector (VICE) genes [46] from Vi1 were identified in the Vi1 WGS. However, the majority of the predicted gene models associated with these loci were longer than those identified using ESTs [46]. One *VICE* gene (*VICE* 13) did not correspond with any AUGUSTUS gene prediction. Only six VICE predicted proteins were included in the secretome of Vi1 (corresponding to *VICE* 4, 5, 6, 9, 10 and 11), five of which were in OrthoMCL clusters in the core secretome, with a single predicted protein from each isolate in each cluster. The sixth predicted protein (corresponding to *VICE* 4) belongs to a cluster with members from only Vi1 (19) and Vp (four). Genes encoding four of these proteins were in the *V*IS set, including the *VICE* 4 gene. The remaining nine *VICE* gene models, assigned by AUGUSTUS, did not encode proteins that were predicted to be secreted. Inspection of these loci revealed that, in the majority of these cases, the N-terminus predicted by AUGUSTUS was upstream of the translation start site predicted by Bowen and associates [46]. Thus the gene predictions at these loci need further validation by sequencing full length cDNAs. Most of the *VICE* gene transcripts were also detected by Thakur et al. [21], in a single transcriptome of *V. inaequalis*; however, these authors did not confirm whether these transcripts encoded predicted secreted proteins.

Two cellophane-induced (*Cin*) genes, *Cin1* and *Cin3*, that encode proteins with internal repeats, have been previously identified as being highly expressed during both in vitro growth in cellophane membranes (10 dpi) and infection of Vi1 on apple (five and 10 dpi) [47]. The RNA-Seq data supported the expression levels reported in the previous study for both of these genes. There were Cin1 orthologues in each of the isolates examined forming a single OrthoMCL cluster, whereas Cin3 orthologues were only in *V. inaequalis* isolates, again forming a single OrthoMCL cluster. *Cin1* was one of the most highly expressed genes in the *V*IS gene set, with very high expression in vitro as well as at both infection time points.

Two additional genes, which encode smaller cysteine-rich Cin1-like proteins, Cin1L1 and Cin1L2 (106 and 169 aa) [48, 49], are also found in each of the isolates. However, their sequence is more variable since a single cluster with representative proteins from each isolate was not observed. The proteins similar to Cin1L1 from each *V. inaequalis* isolate clustered together, but that from the *V. pirina* isolate was classified as a singleton. There are proteins similar to Cin1L2 in each isolate; however, those proteins encoded by the Vi1.10 and Vi1.2.8.9 genomes, although having a signal peptide, were classified as being targeted to the mitochondria by both ProtComp and WoLF PSORT, and the proteins from the remaining isolates formed a single cluster. *Cin1L1* and *Cin1L2* are in the *V*IS gene set.

Of those proteins originally classified as SSPs in the *Venturia* secretomes four shared similarity to proteins in PHI-base [35]: the hydrophobin MHP1 from *Magnaporthe oryzae* [50]; NPP1 from *Hyaloperonospora arabidopsidis* [51–53]; GAS1 from *Magnaporthe oryzae* [54]; BEC1019 from *Blumeria graminis* f. sp. *hordei* [55, 56]. None of these proteins were members of the *V*IS set, although all had evidence of transcription in vitro and in planta (Additional file 12).

#### SSP families in the *V*IS set

**Fig. 5:**
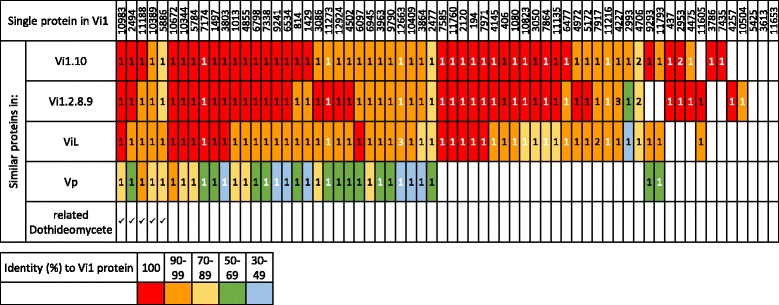
Small secreted proteins (SSPs) in the *Venturia inaequalis* Vi1 secretome encoded by single genes. Only SSPs ≤200 amino acids in length are included. The number of similar proteins in the *Venturia* and related Dothideomycete proteomes are indicated by numbers: black indicates a gene predicted by AUGUSTUS, white indicates a putative coding sequence identified by tBLASTn using the protein sequence as query, followed by manual curation. Percentage identity is represented by: *red* = 100%; *orange* = 90–99%; *yellow* = 70–89%; *green* = 50–69; *blue* = 30–49%

**Fig. 6:**
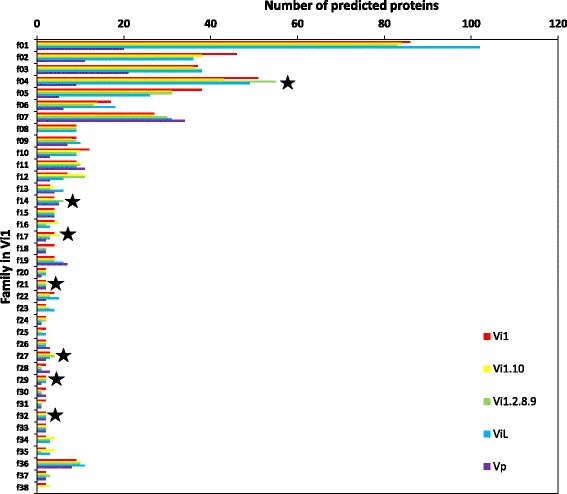
Small secreted protein (SSP) families in four *Venturia inaequalis* and one *V. pirina* secretomes. Only SSPs ≤200 amino acids in length are included. *Red* = proteins in the same family from Vi1; *yellow* = protein families from Vi1.10; *green* = families from Vi1.2.8.9; *blue* = families from ViL; *purple* = families from Vp. Families f1-f28, and f34-f38 are cysteine rich i.e. two or more cysteines per protein; f29-f33 are those with one or no cysteines. ★ = families with predicted similar proteins in related Dothideomycete genomes

Of the 195 *V*IS SSPs ≤200 aa in length in the Vi1 secretome, 64 appear to be single proteins following spectral clustering analysis [57], 121 belong to 38 different families, having between two and 86 members. The families include predicted proteins without in planta up-regulated expression of their corresponding genes and putative proteins that were not predicted by the original gene prediction software (Figs. 5 and 6). The remaining 10 SSPs were not retained in the dataset since, although initially classified as an SSP ≤200 aa in length, they belong to families where the majority of members have a robust annotation to an enzyme based on gene ontology analysis and similarity searches, or are larger than 200 aa in length. Family 4, 6 and 35 are very closely related and as an indication of this these families share members (four are in both family 4 and 6 and one is in both family 6 and 35). A representative sequence logo for family seven is shown in Additional file 13. Although sequence diverse, the families have a putative conserved structure based on conservation of cysteines.

Thirty families of predicted SSPs, and 31 single proteins, have similar proteins present in all of the *Venturia* isolates analysed. All, except five, of the single proteins (from the Vi1 isolate) are also single proteins in all of the isolates studied. Seven families are unique to and conserved within the isolates of *V. inaequalis*, whereas there are 20 single proteins in Vi1 with similar proteins present in all *V. inaequalis* isolates and absent from *V. pirina*. There is one family, and two single proteins, restricted to isolates that only infect apple (Figs. 5 and 6).

In addition, there are four single SSP proteins, and one family, that are putative candidates for the effector/s that contribute to the race profile of isolate Vi1.2.8.9, i.e. present in Vi1 and Vi1.10, but absent from Vi1.2.8.9 (Table 2 and Additional file 14). There is one family and three single proteins that are candidates for effector AvrRvi10, given their presence (in Vi1 and Vi1.2.8.9) and absence (in Vi1.10) in the *Venturia* isolates.

**Table 2:** Small secreted proteins (SSPs) in the *Venturia* infection secretome (*V*IS) set and related fungi

Similar proteins in^a^:						Families in Vi1	Single proteins in Vi1
Vi1	Vi1.10	Vi1.2.8.9	ViL	Vp	Related Dothideomycete		
✓	✓	✓	✓	✓	✓	7	5
✓	✓	✓	✓	✓		23	26
✓	✓	✓	✓			6	20
✓		✓	✓	✓		1	0
✓	✓		✓	✓		0	2
✓	✓	✓				0	3
✓		✓	✓			0	1
✓	✓					1	2
✓		✓				0	2
✓						0	3

Both families and single genes encoding small secreted proteins ≤200 amino acids in length are included. Families were analysed by Spectral Clustering followed by manual curation and analysis of non-predicted putative genes

^a^Ticks in columns indicate presence of a similar protein or proteins in that secretome

To ascertain whether any of the 195 SSPs of the *V*IS set were conserved across the Dothideomycetes, similarity searches (BLASTp) were made against selected Dothideomycete predicted proteomes curated at the JGI (Additional file 15) [58, 59]; this curation includes clustering information pertaining to proteins from each organism. Only SSPs predicted for the Vi1 secretome, which were present in all the *Venturia* isolates, had putative similar members in related Dothideomycete predicted proteomes (Table 2 and Additional file 14, Figs. 5, 6 and 7).

**Fig. 7:**
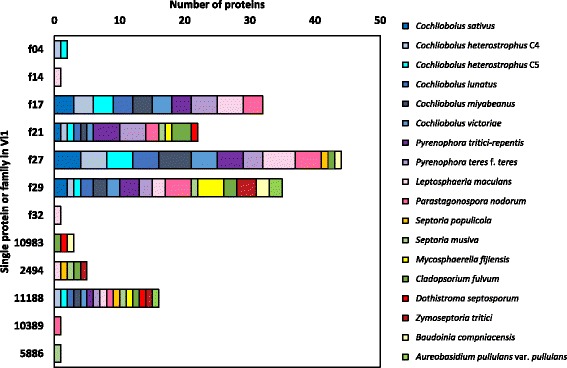
Similar small secreted proteins (SSPs) in Dothideomycete proteomes and the *Venturia inaequalis* Vi1 secretome. Only SSPs ≤200 amino acids in length are included. Shades of *blue*, *purple* and *pink* = Pleosporales: *Cochliobolus sativus*, *C. heterostrophus* C4, *C. heterostrophus* C5, *C. lunatus*, *C. miyabeanus*, *C. victoriae*, *Pyrenophora tritici-repentis*, *P. teres* f. *teres*, *Leptosphaeria maculans*, *Parastagonospora nodorum*; shades of *orange*, *yellow* and *red* = Capnodiales: *Septoria populicola* (teleomorph *Mycosphaerella populicola*), *S. musiva (*teleomorph *M. populorum)*, *M. fijiensis*, *Cladosporium fulvum* (syn: *Passalora fulva*), *Dothistroma septosporum*, *Zymoseptoria tritici*, *Baudoinia compniacensis*; *green* = Dothideales: *Aureobasidium pullulans* var. *pullulans*

#### Putative host range determinants in the SSP set

In addition to those candidate effectors that are specific to either *V. inaequalis*, or those isolates able to infect apple described above, SSPs ≤200 aa in length specific to the loquat-infecting isolate of *V. inaequalis* or to *V. pirina* were identified by OrthoMCL analysis (Additional file 16). In addition to the 62 candidate effectors specific to ViL, only four of which had any similarity to proteins in the NCBI nr database, 6 were found in both the ViL and Vp secretomes. A further 238 candidate effectors were found to be specific to *V. pirina*.

### Gene density and proximity of genes to transposable elements (or transposable element remnants)

**Fig. 8:**
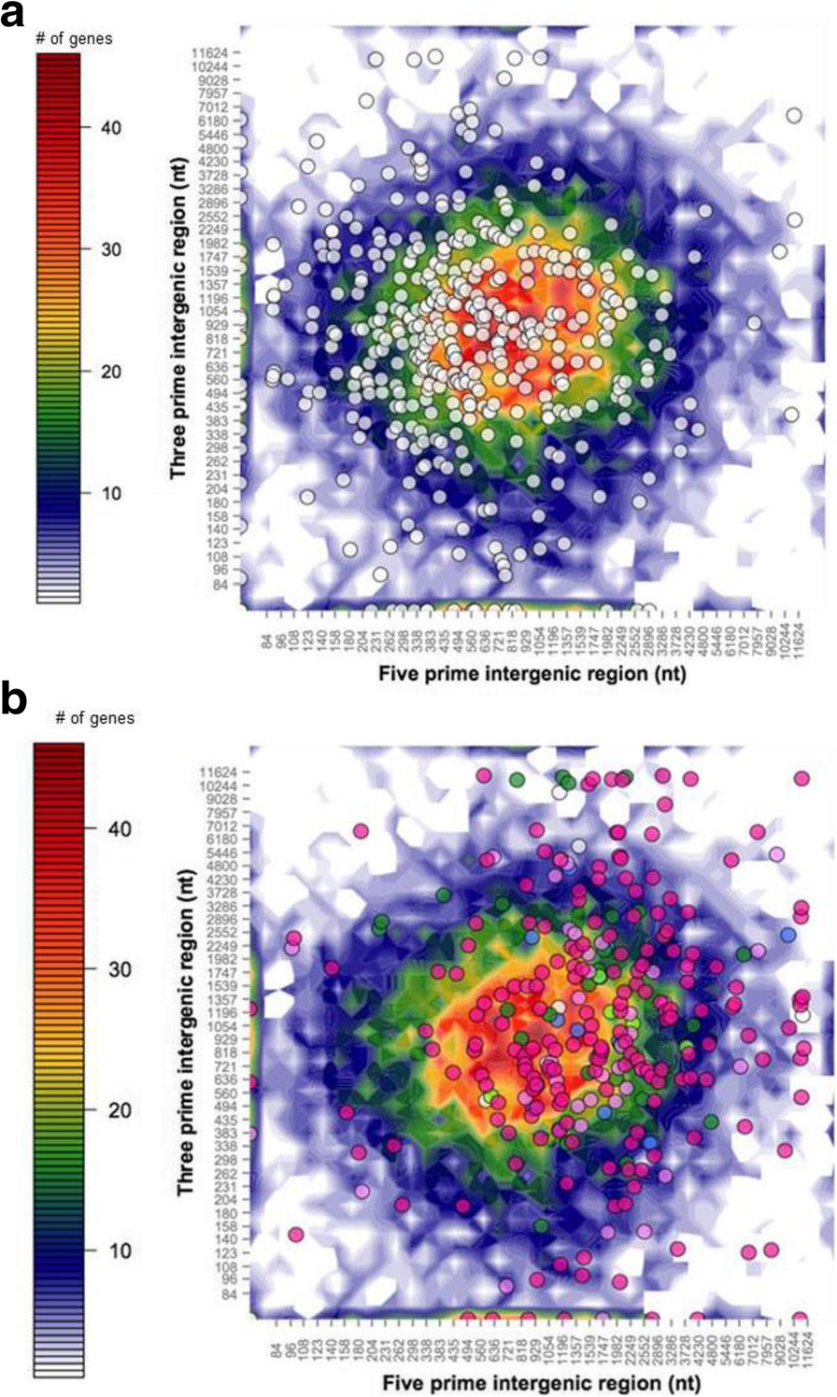
Flanking distance (3′ and 5′) between predicted genes of *Venturia inaequalis* Vi1. Intergenic distances for all predicted genes are represented in the underlying heatmap, with the number of genes in each bin shown as a colour-coded heat map (on orthogonal projection) generated as in Saunders et al. [185]. Genes were sorted into two-dimensional bins on the basis of the lengths of flanking intergenic distances to neighbouring genes at their 5′ and 3′ ends; overlying this are scatterplots of **a** 423 Core Eukaryotic Genes (*white dots*) or **b** *Venturia* infection secretome (*V*IS) gene set, plus *AvrLm6-* and *Ave1*-like genes (coloured dots: *dark pink* = SSPs with two or more cysteines (≤500 amino acids); *light pink* = SSPs with one or no cysteines (≤500 amino acids); blue = peptidases; *dark green* = CAZymes; *light green* = putative cell wall-degrading enzymes (non-CAZyme); *white* = cell wall associated and miscellaneous proteins >500 amino acids). Note that the axes are not linear. Genes at the scaffold end were excluded from this analysis

Most genes in the Vi1 genome have intergenic regions between 200 bp and 3 kb (Fig. 8), with a mean intergenic distance of 1677 bp. A subset of core eukaryotic genes [25] had a slightly shorter mean intergenic distance of 1289 bp, whereas the SSP genes in the *V*IS set (highlighted as pink data points in Fig. 8), display a significantly (*P* < 0.005) longer average intergenic distance at 2560 bp. Genes located on scaffold ends, hence lacking neighbouring genes, were excluded from this analysis. This excluded 17/440 (3.6%) from the core eukaryotic gene set and 21/245 (8.6%) from the SSPs in the *V*IS set. The greater proportion of SSP genes located on the ends of scaffolds suggests a bias for association with complex repeat regions that cause problems with sequencing and assembly, resulting in broken genomic scaffolds.

**Fig. 9:**
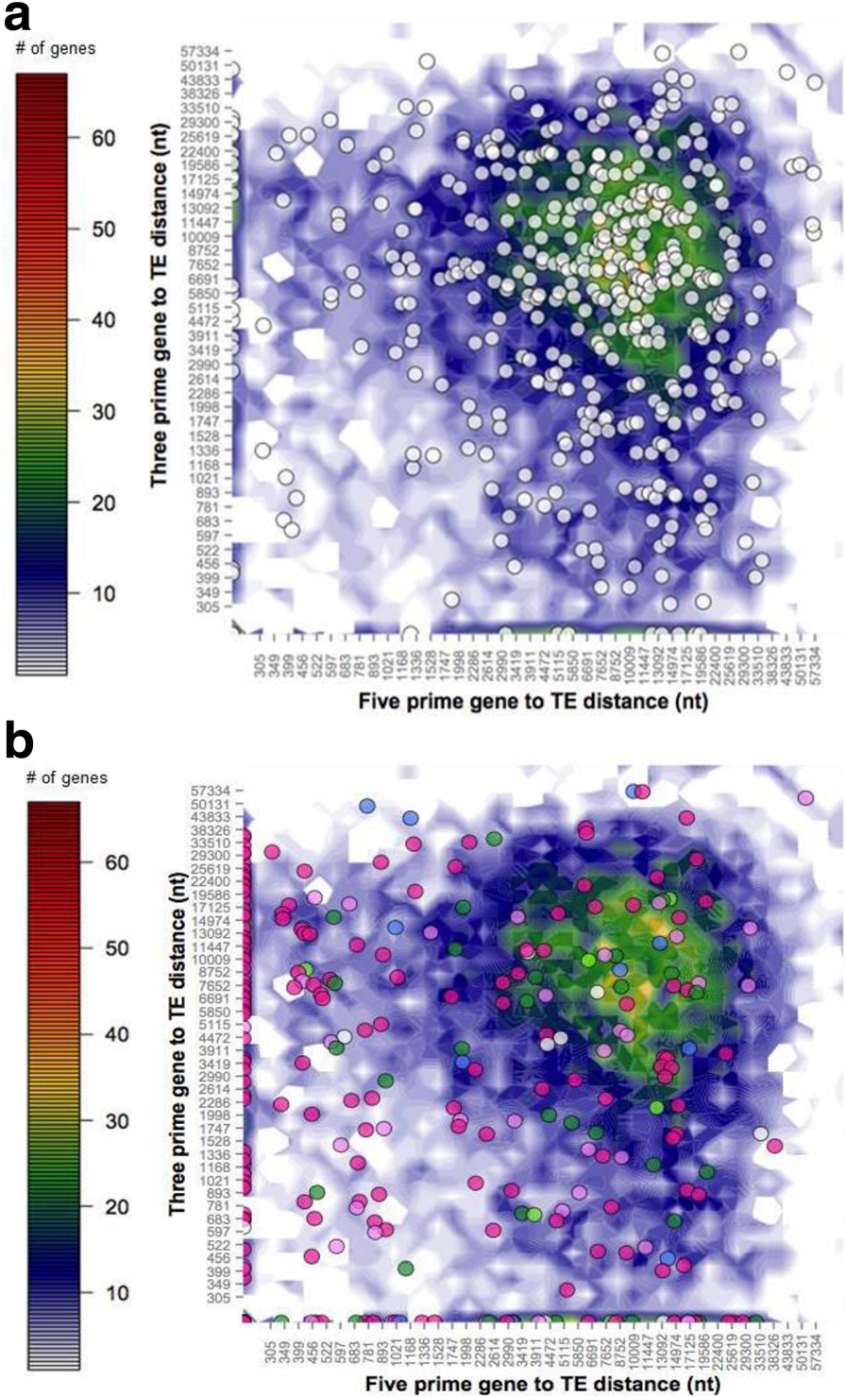
Distance between predicted genes and transposable element (TE)-like features of *Venturia inaequalis* Vi1. Flanking distances (3′ and 5′) to TE-like features for all predicted genes are represented in the underlying heat map, with the number of genes in each bin shown as a colour-coded heat map on orthogonal projection (generated as in Saunders et al. [185]). Genes were sorted into two dimensional bins on the basis of the lengths of flanking distances. **a** 423 Core Eukaryotic Genes (*white dots*) are similar to all genes in that they are not closely associated with TE-like features; whereas **b** *Venturia* infection secretome (*V*IS) gene set, plus *AvrLm6-* and *Ave1*-like genes (coloured dots: *dark pink* = SSPs with two or more cysteines (≤500 amino acid); *light pink* = SSPs with one or no cysteines or less (≤500 amino acids); blue = enzymes (peptidases/redox/primary metabolism); *dark green* = CAZymes; *light green* = putative cell wall-degrading enzymes (non-CAZyme); *white* = cell wall associated and miscellaneous proteins >500 amino acids in length). Note that the axes are not linear. Genes at the scaffold end were excluded from this analysis

Concordantly, SSP genes also appear to be more closely associated with transposable elements (TEs) or TE-like features than core eukaryotic genes and all remaining genes in the Vi1 genome. The mean gene to TE distance was 9024 bp for all genes predicted in the genome (Fig. 9). The core eukaryotic gene set had a significantly (*P* < 0.005) greater mean distance to the nearest TE of 10,528 bp, whereas the SSPs in the *V*IS set had a mean distance to the nearest TE (or TE-like features) of only 5401 bp. Twenty-two SSPs in the *V*IS set were nested within predicted TE-like features. Fourteen of the core eukaryotic genes and six of the *V*IS set SSP genes were excluded from this analysis as they lacked a neighbouring TE feature on the same scaffold.

## Discussion

### Comparisons of whole genome assemblies and gene predictions

This paper reports the first analysis of whole genome sequences from multiple isolates of *V. inaequalis* (*V. pirina* being released in 2014) [22]. The sizes of all five *Venturia* genomes are comparable to those of other Dothideomycetes [60]; however, these may be an underestimate since the majority of the sequencing was carried out on the Illumina platform. Assembling repeat regions using short reads is notoriously difficult [61–64], and hinders genome assembly using de Bruijn graph-based genome assemblers like Velvet and ALLPATHS-LG [62, 64, 65].

In addition, there is an apparent wide range of genome size that can be largely attributed to additional repetitive content, with the assembled genic regions being similar in size for all five *Venturia* isolates. Whilst variation in genome size between isolates from the same species is not unprecedented; for example, genome size in three isolates of the Dothideomycete *Zymoseptoria tritici* (synonym *Mycosphaerella graminicola*) ranged from 31 to 40 Mb as estimated by pulsed field gel electrophoresis [66], this apparent variation in the *Venturia* genomes may be attributable to the different strategies, and software, used for sequencing. The genomes (Vi1.10 and ViL) assembled using ALLPATHS-LG software, have much larger genome sizes and higher percentages of repeats, than those assembled using Velvet (Vi1, Vi1.2.8.9, and Vp). The major difference between the sequencing of these five isolates is that Vi1.10 and ViL paired-end (PE) libraries were specifically constructed with an insert size of 180 bp so that paired reads overlapped at the end (which is required by ALLPATHS-LG), to produce longer super-reads. For the other three isolates, the PE libraries were constructed with a typical insert size of 400 bp, and paired reads did not overlap and could not be assembled using ALLPATHS-LG. The super-reads generated by ALLPATHS-LG result in repeats being better assembled [67, 68]. As a trial, in addition to ALLPATHS-LG, the Vi1.10 and ViL sequences were also assembled using Velvet. The percentage of repeats in the Velvet scaffolds for each isolate was lower than that for the ALLPATHS-LG assembly, but was still very high compared with the remaining three isolates, with 21.5 and 25% for Vi1.10 and ViL respectively. Additional investigation is required to determine whether the larger genome sizes and higher percentages of repeats in Vi1.10 and ViL are a biological reality.

The number of predicted genes in the *Venturia* genomes (11,960–13,333) is significantly less than the 24,571 unique fungal genes reported for *V. inaequalis* by Thakur et al. [21]. Just over half of the reported sequences had no significant similarity to sequences in other species. The Thakur et al. [21] estimate was based on in vitro and in planta transcriptome data only, with multiple gene splice variants likely to account for the higher gene number. Our estimate of predicted gene number is comparable to related Dothideomycete genomes which range from 9739 in *S. populicola* [60] to 14,127 in *C. fulvum* [69]. Gene number variation could be due to difference in coverage of the various genomes; however, variation in gene number between isolates is also not unprecedented. For example, Xue and associates [70] found that in the rice blast fungus *M. oryzae*, gene number varied, with hundreds of isolate-specific genes present in genomes of field isolates. Similar gene number variation was reported in isolates of *C. heterostrophus* [71].

### *Venturia* pathogens: lifestyle and host determination

Focussing on the secretome of the *Venturia* pathogens has revealed repertoires of proteins that reflect aspects of their adopted mode of parasitism, with secretion of compounds to adhere to the waxy, water-proof cuticle on leaves and fruit and to enable direct penetration and colonisation of the cuticle and sub-cuticular space. In addition, effectors that are predicted to effect evasion of recognition and suppression of host defence will be secreted to the extracellular plant-pathogen interface, including those that may be directed to be taken up by the host cells.

#### Attachment and penetration

Several *Venturia* proteins were identified in the secretomes that may be involved in attachment and penetration of the cuticle. A predicted protein present in all the Dothideomycete genomes analysed has similarity to a *M. oryzae* fasciclin protein. Fasciclins have been implicated in cell adhesion in diverse organisms including both prokaryotes and eukaryotes [72–75]. The *M. oryzae* fasciclin has an important role in development and pathogenicity, involved in conidiation and conidial adhesion [76]. A similar role for fasciclins in cell adhesion for all the *Venturia* isolates analysed is easily envisaged.

Hydrophobins are small, secreted, cysteine-rich, amphipathic proteins that are usually found on the cell walls of fungi [77, 78]. They provide a water-repellent coat and are well characterized for their role in morphogenesis and virulence in plant pathogenic fungi, promoting interactions with hydrophobic surfaces [40, 41, 79, 80]. It is thought that these proteins may also assist in avoiding detection by the host during infection [77]. Hydrophobins are usually highly variable in sequence; however, they have a conserved structure due to conserved cysteine residues. *Venturia* secretomes have fewer hydrophobin genes (two-to-four) than *C. fulvum* (11) but have a similar number to *D. septosporum* (four) [69].

The *Venturia* secretomes also have a high number of genes (11–14) encoding putative HsbA proteins. HsbA proteins are found in entomopathogenic fungi as well as pathogens of higher animals; with cuticle colonisation being a possible common link. HsbAs are not commonly reported to be encoded by plant pathogen genomes (although genes with low similarity to *V. inaequalis HsbA*-like genes were identified in genome sequences of members within *Colletotrichum*, *Magnaporthe* and *Verticillium* genera). One sequence with similarity to an HsbA was identified in the genome of the mesophyll apoplast-dwelling *C. fulvum* and the pine pathogen *D. septosporum* [69]. HsbAs are not hydrophobins, but they have been reported [81] to have an analagous role in *A. oryzae* in binding to hydrophobic surfaces and recruiting the CutL1 polyesterase/cutinase to degrade the polyester substrate, poly(butylene succinate-co-adipate) (PBSA). Takahashi and associates [82] also reported that the *A. oryzae* hydrophobin, RolA, also recruits CutL1, to aid degradation of PBSA surfaces. Thus, *A. oryzae* appears to use several types of proteins to recruit lytic enzymes to the surface of hydrophobic solid materials and promote their degradation. Proteins similar to the *A. oryzae* CutL1 polyesterase/cutinase and HsbA were well represented in the *V*IS set. Extracellular cutinase has been implicated in penetration by *V. inaequalis*, as cutinase is produced by germinating conidia, and a cutinase inhibitor can prevent penetration [83, 84]. In addition, esterase-like activity has been reported during the germination of conidia and in appressoria [85]. The presence of high numbers of CEs in the *Venturia* secretomes supports the experimental evidence of enzymatic penetration of the cuticle by *V. inaequalis* and the inference that *V. pirina* also may act similarly. We propose that *Venturia*, like *A. oryzae*, uses multiple proteins (e.g. hydrophobins and HsbA proteins) to recruit cutinases/esterases to facilitate appressorial adhesion and direct penetration, as well as degradation and digestion of cuticle. This hypothesis fits observations of the cuticular degradation that occurs during colonisation by *Venturia* fungi potentially drawing nutrition from the cuticle or cuticle precursors [7].

#### Nutrition

Exploitation of the cuticle may be insufficient to satisfy pathogen nutritional requirements throughout the infection cycle. During biotrophic infection *Venturia* remains in the cuticle and sub-cuticular space causing relatively little damage to the host. Most damage is due to breaching of the cuticle upon sporulation [6, 7]. At this time the epidermal cells underlying the stroma undergo a progressive depletion of plastids and cytoplasm, accompanied by increasing vacuolation, leading ultimately to cell death. It has been proposed that this damage late in the infection cycle is caused by partial cell wall degradation [86, 87]. Indeed, cellulase and pectinase activities have all been reported for *V. inaequalis* growing in vitro [6, 88, 89]. However, it has been suggested that the timing of this host cell degradation precludes a significant role for these enzymes in nutrient acquisition [89, 90].

The predominant polysaccharide in apple fruit skin is pectin at 65%, compared with a level of 3% for cellulose [91], with pear fruit also having higher levels of pectin than cellulose [92]. *Venturia* appears to have tailored its CAZyme repertoire to suit the composition of the host cell wall with pectin-specific CAZymes predominating; the two most numerous classes of CAZymes found in the *Venturia* secretomes, after those with cutinase activity, are GH28 and GH43 that have pectin as a substrate. A single GH28 enzyme and two putative PLs, that also have pectin as target substrate [31, 93], are up-regulated during infection and are present in the *V*IS set. Significantly, this lytic activity may therefore not be limited to late in the infection cycle as previously thought, but contribute to nutrition via degrading the surface polysaccharides of the epidermal cells beneath stromata, this damage only becoming evident macroscopically late in infection. In addition, those CAZymes present in the secretomes of the *Venturia* fungi may also benefit the fungus during saprobic growth and sexual fruiting body development during winter.

#### Evasion of host defence

The putative proteases present in the secretomes of the *Venturia* fungi may aid in the evasion of host defense since proteases have been implicated in plant defence avoidance in many plant-pathogen interactions [94]. For example, fungal proteases can target plant chitinases, as in the interaction between *Fusarium oxysporum* f. sp *lycopersici* and tomato where both a serine protease and a metalloprotease are required for the inactivation of a chitin-binding domain (CBD)-containing chitinase thus contributing to virulence [95].

As a possible alternative strategy to counteract host chitinases the *Venturia* strains, in common with many other fungi, all have a homologue of the *C. fulvum* LysM domain effector, Ecp6. These are likely to either have a similar function to *C. fulvum* Ecp6 [42, 96] in preventing recognition of chitin fragments capable of initiating a defence response, or to the LysM effectors from *Z. tritici* that protect hyphae from the action of chitinases, thus preventing the release of chitin-derived PAMPs [97, 98]. In addition, there are single proteins in each secretome similar to the *Blumeria* effector BEC1019 [55, 56]. The putative *Venturia* BEC1019 orthologues all have the conserved ETVIC motif that is required for suppression of the hypersensitive response (HR) in barley. A similar function for these proteins during the pathogenicity of *Venturia* is therefore easily envisaged.

Although single proteins similar to the NPP1 protein from *H. arabidopsidis* are present in each of the *Venturia* secretomes [51, 52] the sequence of these from two apple-infecting isolates are truncated. In the remaining secretomes (Vi1, ViL and Vp) the NPP1-similar proteins appear to belong to the type I Nep-1-like protein (NLP) family, with the two conserved cysteines [99]. All three proteins also have two additional C-terminal cysteines, however these are not characteristic of type 2 NLPs [100]. The majority of NLPs induce necrosis in a wide-range of dicotyledonous plants, although whether this necrosis is a direct result of cytotoxicity via dispruption of the plasma membrane or initiation of a defence response remains open to debate [100]. However, the requirement for a functional necrosis-inducing protein in the biotrophic *Venturia* pathogens is unlikely. Indeed, each of the *Venturia* proteins has mutations in the critical H residue in the loop region required for necrosis in the NLP from *V. dahliae* [101], whilst the two *V. inaequalis* proteins also have a mutation in the heptapeptide domain (GHRHDWE mutated to GHRHEWE) required for necrosis in NLPs from diverse taxa [52, 100, 102]. These mutations may therefore prevent unwanted pathogen-induced necrosis, but the retention of these NLP-like proteins by *Venturia* may indicate a virulence function that remains to be elucidated.

*Venturia* predicted proteins with similarity to the *Magnaporthe* GAS1 protein, required for appressorial penetration and lesion development, may play a similar role in apple and pear scab diseases to that observed in rice. Whether GAS1 is translocated to the host cytoplasm with a role in defence suppression remains equivocal, however; weak fluorescence of fluorescent protein constructs in infectious hyphae in onion epidermal cells was observed [54], as was translocation to rice cytoplasm, albeit under, presumably, the heterologous protomoter from the *Magnaporthe* gene *PWL2* [103]. The *Venturia* proteins may therefore be translocated to the host cell cytoplasm where they may have a role in re-directing host metabolism during scab disease, however this remains conjecture.

#### Initiation of host resistance: an avirulence function

The comparative approach undertaken here has enabled preliminary identification of candidates for *V. inaequalis* avirulence effectors AvrRvi2, AvrRvi8, AvrRvi9 and AvrRvi10, which have an avirulence function and are recognised by cognate R proteins. All candidates have cysteine residues but no recognisable protein motifs, except a single candidate for AvrRvi2, AvrRvi8 or AvrRvi9 that is similar to a bacterial chaplin with a domain of unknown function, that may play a role in the *Venturia* lifecycle similar to that of hydrophobins. In addition to SSPs that may be involved in race/cultivar specificity on apple within *V. inaequalis*, further SSPs were identified that are specific to the loquat-specific isolate of *V. inaequalis* or to *V. pirina*. The majority of these SSPs were either similar to hypothetical proteins available in the public domain or had no similarity with known proteins. However, of interest is a protein with ankyrin repeats that may be involved in the specificity of *V. pirina*, since it is dissimilar to proteins found in the other secretomes. Ankyrin repeat-containing proteins are involved in protein-protein interactions and are present in effectors from both prokaryotes and eukaryotes, and as such this protein may play a role in the interaction with European pear [104]. In addition, a single protein specific to the ViL isolate has a SnoaL-like domain. SnoaL-like domains are found in proteins from diverse taxa, including filamentous fungal phytopathogens. For example, the protein PEP2 from *Nectria haematococca* MPVI has such a domain and contributes to virulence on pea; complementing an isolate of *N. haematococca* lacking a supernumerary chromosome bearing pathogenicity genes with *PEP2* results in a small, but significant, increase in virulence [105]. How these candidate effectors may be contributing to host range determination is unknown. They may be acting as avirulence proteins during attempted infection of a nonhost, with a recognition event triggering resistance, and a concomitant lack of recognition in a compatible host. Indeed, since the hosts are closely related [106], the contribution of R protein-triggered immunity towards nonhost resistance has been postulated to be greater than for host plants that are more distantly related [3]. The variability of the closely related genomes of the *Venturiaceae* with respect to SSPs reinforces the utility of a comparative genomic approach.

In each of the *V. inaequalis* secretomes the predicted proteins with similarity to AVR-Pita1 from *M. oryzae* may play a similar role to that adopted by both AVR-Pita1, and its paralogue AVR-Pita2, in the rice blast interaction [107, 108]. Although the virulence function of AVR-Pita1, a putative zinc metalloprotease, has not been verified it is directly recognised by the cognate *R* gene product Pi-ta. An avirulence function for the similar proteins in the *Venturia* pathogens remains to be elucidated.

#### Effectors with unknown function

In addition to those proteins with similarity to known effectors with roles partially or fully confirmed, the *Venturia* secretomes comprise a large repertoire of putative effector SSPs with no obvious hints as to function from similarity searches. The paradigm for the role of SSPs is that they are lineage-specific effectors that contribute to the maintenance and evolution of parasitism and that obligate biotrophy is associated with expanded effector repertoires [109, 110]. It is thought that biotrophs require a more extensive and nuanced effector inventory than that required by necrotrophs, that rely more prominently on cell-wall degrading enzymes and toxins [111]. Although this précis of fungal pathogen lifestyles is highly simplistic, it seems to be reflected in the fewer number of SSPs recorded for necrotrophic fungal phytopathogens. For example *Parastagonospora nodorum* has a suite of only 209 SSPs [60], and a recent analysis of the secretome of *Sclerotinia sclerotiorum* revealed only 78 effector candidates, albeit using different criteria for selection [112]. This contrasts with the numbers of SSPs reported for the obligate biotrophs: *B. graminis* (490) [113], although these are up to 400 aa in length; *P. graminis* f. sp. *tritici* (540); and *Melampsora larici-populina* (305) [60]. These studies use different criteria for SSP identification, hampering valid comparsions. In the current study, SSPs from *P. nodorum*, *C. fulvum* and *P. graminis* f. sp. *tritici* were predicted using the same pipeline as that used for SSP analysis from the *Venturia* pathogens*.* The number of SSPs in the *Venturia* pathogens were between that recorded for the obligate biotroph *P. graminis* f. sp. *tritici* and the necrotroph *P. nodorum* or the facultative biotroph *C. fulvum*. For those SSPs predicted to be less than 200 aa in length, the proportion predicted to be classically secreted predominated. This profile resembled that of *P. graminis* f. sp. *tritici*. Thus overall the repertoire of SSPs from the *Venturia* pathogens more closely resembles that of an obligate biotroph rather than a necrotroph or facultative biotroph.

Evidence of high expression or up-regulation during infection (compared with in vitro growth) was used to prioritise a smaller set of *Venturia* SSPs [114]. The smallest of these uncharacterised SSPs were analysed in more detail and of those with similarity to predicted proteins in related Dothideomycete genomes, only four were found in the majority of genomes investigated. These Dothideomycete genomes are from organisms with diverse lifestyles. Up until recently this broad conservation would indicate a core metabolic role, rather than a role in pathogenicity, especially for those proteins also found in the extremophilic sooty mould saprobe, *Baudoinia compniacensis*, with its compact genome of 21.88 Mb [60]. However, the recent research of Whigham and associates [56] showing that the broadly conserved effector BEC1019 from the pathogen *Blumeria*, that can suppress HR, may be repurposed to fulfil particular roles in fungi with diverse lifestyles, challenges this view.

A small family of proteins in *V. inaequalis* with similarity to Bys1 from *M. oryzae* also has representatives in other diverse Dothideomycetes. The function of the Bys1 domain is unknown, but in *Blastomyces dermatitidis* the expression of a Bys1-encoding gene is associated with pathogenesis [115, 116], thus a similar role in Dothideomycete pathogens cannot be ruled out.

One of the most highly expressed genes in the *V*IS gene set, with expression in vitro and in planta, encodes a protein with similarity to the *Alternaria alternata* major allergen Alt a 1 (GenBank: AAM90320.1). This *V. inaequalis* protein was identified previously [117] in an analysis of semi-purified secreted proteins that elicited a response from specific resistant apple hosts. Single genes encoding proteins with similarity to the Alt a 1 allergen were identified in all *Venturia* genomes and a single gene was also identified in some (but not all), related Dothideomycetes (*P. nodorum; S. populicola*, *S. musiva*, *C. fulvum*, *Z. tritici*). Alt a 1 is well known in clinical settings as a human allergen. It has a structure unique to fungi; however, its role in pathogenesis has not as yet been determined [118].

A large number of SSPs in the *V*IS set with similar proteins across Dothideomycete spp. have an internal repeat structure. Repeat structures in fungal effectors have been reported previously [104]. For example the SP7 effector from *Glomus intraradices*, an arbuscular mycorrhizal biotroph, has nine hydrophilic tandem repeats. SP7 re-programmes plant expression to reduce the defence response thus enabling establishment of the fungus within the roots of plants [119]. In addition, a double knock-out mutant of *U. maydis* lacking the repeat-containing protein effectors, Hum3/Rsp1, has arrested growth *in planta*, shortly after penetration [120]. The roles of the previously identified repeat-containing proteins, Cin1 and Cin3, which are expressed very highly in the stromata and runner hyphae of *Venturia*, are yet to be determined [48].

#### Expanded SSP effector families

Intriguingly, *Venturia* strains have multiple genes predicted to encode proteins with similarity to AvrLm6 from the Dothideomycete *L. maculans* and Ave1 from the Sordariomycete *Verticillium* spp. These effectors are encoded by a single gene (*Ave1*) in *Verticillium* spp. [44], or a single gene plus a paralogue (*AvrLm6*) in *L. maculans* [121].

The *Venturia Ave1*-like genes were generally not found in clusters on contigs and both effector families have significant numbers of pseudogenes (lacking a start and/or a stop codon). The role of these Ave1-like proteins is yet to be determined; however, the multiple gene members in the *AvrLm6* and *Ave1* effector families will present a challenge for functional analyses. *AvrLm6*-like genes are not found in most of the sequenced Dothideomycete relatives of *L. maculans*. The same is true for *Ave1* from the Sordariomycete, *Verticillium*. This discontinuous distribution of *Ave1* and *AvrLm6* suggests that these effectors are either ancient effector ancestors that have been lost or diversified beyond recognition during coevolution and host specialisation, or that there may have been horizontal gene transfer events involving the *Ave1*- and the *AvrLm6*-containing species. None of these species share a common host, however, and so mechanisms for gene transfer are not obvious.

Many of these *Venturia* effector Ave1 and AvrLm6 orthologues were not identified by the secretome prediction pipeline as their N-terminus was incorrectly designated by the automated gene calling software packages. Ave1-like proteins have also been detected by Mass Spectrometry in an analysis of *V. pirina* proteins expressed in vitro [22]. The presence of a signal peptide, mature N terminus, cleavage site and presence of a conserved intron in the 5′ UTR of the gene, was confirmed for several of the *V. pirina* Vp *Ave1*-like genes in this proteogenomic analysis. The conserved intron in the 5′ UTR of *Ave1-*like genes appears to have caused difficulties in accurate gene prediction for all genes in this family. Rigorous interrogation of the whole set of SSPs, with a size of 200 aa or less, also highlighted the challenges of gene prediction. On initial comparison of gene predictions, via comparison of their encoded proteins, genes encoding SSPs similar to those in the Vi1 secretome were not predicted in the other genomes. To verify this absence, comparison of aa sequence against a six-frame translation of the genomes was undertaken and revealed the presence of identical or near-identical loci. The use of RNA-seq data to inform gene prediction in the Vi1 genome appeared to be more efficient than using a model based upon these data for gene prediction in the other genomes, highlighting the importance of RNA-seq data for individual genomes for accurate gene prediction.

With the exception of the families comprising *Ave1*- and *AvrLm6*–like genes, the majority of families of SSP genes in *Venturia* do not have similar genes in the other Dothideomycete genomes analysed. These families appear to be lineage-specific. Lineage-specific genes are common features of fungal genomes sequenced to date [122]. In the *Venturia* pathogens many of the lineage-specific SSPs appear to belong to expanded families, for example, eight SSP families with members of less than 200 aa have more than 10 members, and up to 86 members, and are restricted to the *Venturia* genus. Gene gain and also expansion of these lineage-specific families is obviously associated with host range determination and specificity, and the converse, reduction or loss of gene families can likewise be associated with evolution of virulence on a particular host [122]. Expansion of lineage-specific effector families has recently been reported in *B. graminis* [123], with the 1350 paralogous copies of *AVR*_*k1*_ and *AVR*_*a10*_, that contribute to the establishment of the haustorium, being an extreme example [124, 125].

Whether the members of the SSP gene families observed in the *Venturia* genomes are derived from a common ancestor remains equivocal since their overall sequence conservation is low. However, de Guillen et al. [126] identified a family of sequence-unrelated, but structurally conserved effectors (termed MAX-effectors) in *Magnaporthe*, accounting for 5–10% of the effector repertoire. These effectors have been presumed to have evolved via diversifying selection rather than convergent evolution [126]. In addition, hydrophobins, with their patchy distribution and low level of sequence conservation, contrasting with a highly conserved structure, related to function, appear to evolve by a birth-and-death mechanism and to be phylogenetically related [127, 128]. The families of *Venturia* SSPs with retention of presumably critical cysteines, in terms of structure and stability, may have also arisen under similar evolutionary constraints and thus also be related phylogenetically. Evolution of protein families involves the duplication of an ancestral gene followed by mutation of the duplicated gene to enable novel functionality to emerge [129, 130]. In the case of effectors, expansion and diversification within a family may enable modification of function to prevent alerting a guarding resistance gene product, or structural modification to avoid a direct recognition event that would otherwise elicit a defense response. Thus having expanded families of effectors may offer an evolutionary selective advantage to a pathogen, enabling recognition of an effector to be overcome with evolution of a novel paralogue and deletion or pseudogenization of the previously recognised effector. The processes involved in how these families expand is still open to debate. The presence of large numbers of presumably structurally related, but sequence diverse, proteins poses problems for the delineation of families. Indeed, three families were delineated in this study that shared common members. Although an unorthodox compromise, these families were deemed to be too diverse to be amalgamated using the thresholds adopted for this analysis, but the members are sufficiently closely related to form a single family if the thresholds were to be slightly relaxed, thereby highlighting the pitfalls of depending on arbitrary thresholds for either separating or grouping proteins.

In many pathogens, effector genes are associated with TEs. Association of TEs is evident with the co-evolution of the *B. graminis AVR*_*k1*_ effector family with a class of LINE-1 retrotransposons [124], the *B. graminis* AvrPm3a2/f2 effector family with various TEs [123] and the association of miniature impala transposons (mimps) in the promoters of effectors in *F. oxysporum* [131]. TEs have also been implicated in horizontal gene transfer [132]. The analyses of gene density, and association of effector candidates with repeat elements in Vi1, suggests that effectors and TEs may be similarly associated in *Venturia*. In addition, most of the *AvrLm6-* and *Ave1*-like genes are closely associated with repeats, i.e. TEs and TE-like remnants, as identified by REPET [45]. Oliver [133] outlined the mutagenic potential of transposons and their, at first, seemingly unlikely contribution to the evolution of a successful pathogen: transposons contribute to diversity generation by insertion either in or near a gene, affecting either structure of the resulting encoded protein or the expression pattern, respectively. Transposons may facilitate gene family expansion through capture and translocation of host genes [134], such expansions can become targets for repeat induced point mutation (RIP), with mutations occuring not only in the duplicated transposons but the associated genes [133, 135, 136]. This is an ongoing area of interest and analysis of repeat regions is currently underway in cross progeny of *V. inaequalis*. Further analyses are required in the *Venturia* spp. to reveal if TEs and indeed RIP are involved in diversity generation, and expansion of gene families as suggested by this initial analysis.

## Conclusions

The comparative analysis of whole secreteomes of multiple races of *V. inaequalis* with the related scab pathogen, *V. pirina*, has provided novel insights into the unusual, biotrophic lifestyle niche that these pathogens occupy. It has also yielded significant leads in the hunt for cultivar- and host-specificity determinants of scab fungi. The challenge now will be to prioritise leads, from the expanded arrays of putative effectors, for futher investigation with a mind to delivering durable, scab control.

## Methods

### Fungal material and culture conditions

All *V. inaequalis* and *V. pirina* isolates used in this study (Table 1; Additional file 17) have been reported previously [12, 22, 46, 137, 138] and morphology, pathology as well as DNA sequence comparisons (ribosomal RNA ITS1-5.8-ITS2 and TEF1) have been used to validate the classifications of these isolates as *V. inaequalis* or *V. pirina.* All isolates were grown on cellophane (Waugh Rubber Bands, Wellington, New Zealand) [139] overlying potato dextrose agar (PDA) at 20 °C for 18 h to 14 days (16 h light period/day) under white fluorescent lights (4300 K) for the production of conidia for plant inoculation (Vi1 only) and biomass for genomic DNA (gDNA; all isolates) and RNA extraction (Vi1 only). Cellophane was used, as it induces the formation of spores and stromata-like tissues in *Venturia* spp*.* similar to those formed during infection [47].

### Plant material and infection assays

Four- to six-week-old seedlings originating from open-pollinated *Malus* x *domestica* ‘Royal Gala’ (Hawke’s Bay, New Zealand) were used to produce infected plant material. A detached leaf assay was used for generating tissue for RNA sequencing [117], except that 5 μl droplets of conidial suspension (1 × 10^5^ ml^−1^) were used to cover the entire leaf surface. *V. inaequalis* Vi1-infected and SDW-inoculated leaves were harvested at two or seven dpi and used for RNA extraction and microscopic evaluation of infection.

### gDNA extraction and whole genome sequencing

Extraction of *V. inaequalis* gDNA was carried out as reported in Kucheryava and associates [47]. Two PE libraries and two mate-pair (MP) libraries (with 5 kb and 10 kb insert sizes) were constructed for Vi1. These libraries were sequenced on the HiSeq2000 platform at the Allan Wilson Centre Genome Service (AWCGS), Massey University, New Zealand, and the Australian Genome Research Facility (AGRF). One plate of a whole genome library was also sequenced by the AWCGS on the Roche 454 Life Sciences Genome Sequencer with read length ranging from 18 to 634 bases. One PE and one MP library were constructed for each of the isolates, Vi1.10 and ViL. The PE libraries were specially built with a fragment size of 180 bp so that read 1 and 2 overlapped at the end. All four libraries were sequenced on the Illumina HiSeq2000 platform at Macrogen, Korea. The Vi.1.2.8.9 PE library was sequenced in one lane on the Illumina HiSeq2000 at the AGRF. Genome sequencing details for Vp were reported in Cooke et al. [22]. The majority of the genomes were sequenced using the Illumina platform, apart from Vi1 which is a hybrid assembly of reads from both Illumina HiSeq and Roche 454 platforms.

### RNA extraction and transcriptome sequencing

Total RNA was extracted by the method of Chang et al*.* [140] and concentration quantified using a Nanodrop, ND-1000 Spectrophotometer (NanoDrop Technologies, Rockland, DE). For RNA sequencing cellophane membranes were harvested as follows and combined for a single RNA extraction: three cellophanes 18 h post inoculation (hpi), four cellophanes four dpi, four cellophanes six dpi and one quarter of a cellophane 14 dpi, stripped of aerial hyphae (to enrich for stromatic tissues inside the cellophane sheet). For in planta material three detached leaves inoculated with *V. inaequalis* conidia (detached leaf assay) were harvested at two and seven dpi and snap frozen in liquid nitrogen. RNA was extracted as above, a single sample being derived from the three leaves. Genomic DNA contamination was excluded by visualisation of RNA on a 0.8% agarose gel and absence of an amplification product using primers specific for glyceraldehyde 3-phosphate dehydrogenase genes from *V. inaequalis* and apple [47]. The RNA from in vitro and in planta extractions (5 μg), from the two different time points, was sequenced on the Illumina HiSeq2000 platform in PE mode AGRF, giving between 17 and 33 million pairs of reads per sample.

### Bioinformatic analyses

#### Genome assembly

For each *Venturia* isolate, a quality check (QC) on genome sequencing data was carried out utilising PIQA v1.0 [141], the FASTX toolkit v0.0.13 [142] and FastQC [143]. Reads were trimmed using in-house PERL tools and adaptor sequences were removed using fastq-mcf [144] based on data QC reports. De novo genome assembly was performed using Velvet [61] and SOAPdenovo with a range of Kmer values for Vi1 and Vi1.2.8.9 [145]. For each isolate, the top three assemblies that produced the best statistics in terms of size of contigs and scaffolds, and N50 and N90 metrics, were chosen for futher analysis. CEGMA [25] and BUSCO [26] tests were performed on various genome assemblies to check their completeness; utilising the 248 most conserved core genes for the former [146]. Known *Venturia* ESTs (ABEA, ABEB, IAAA, MAAB and MAAD libraries deposited as ESTs at NCBI) [46] were aligned to the genome assemblies for quality evaluation i.e. to ensure correct assembly of the corresponding sequences in the genome using a BLASTn similarity threshold of <1e-05. The assembly with the highest percentage completeness measured by the CEGMA analysis and the best-mapping ESTs was selected as the genome to release for the isolate. Isolates Vi1.10, and ViL, were assembled with three genome assemblers, Velvet, SOAPdenovo, and the ALLPATHS-LG programme since their PE libraries were constructed specifically with overlapping pairs and satisfied the requirements for ALLPATHS-LG [62, 64]. As for the other isolates, the assembly with the best statistics was chosen for further improvement. The best genome assembly for each isolate was gapfilled using GapCloser with PE reads (GapCloser in SOAPdenovo version 1.12) [145] then scaffolded using SSPACE with MP reads (SSPACE version 2) [147]. The gapfilling and scaffolding steps were performed iteratively until there was no obvious gap size decrease or reduction in scaffold number.

#### Gene prediction

The assembled genomes were masked before gene prediction using a customized pipeline which included RepeatMasker-open-3-3-0, RepBase [148], RepeatScout [149], trf [150] and TEClass [151]. Fungal genes downloaded from NCBI (August, 2012) were mapped to the Vi1 genome assembly using Exonerate [152] for similarity based gene prediction. Transcriptome assemblies for in planta and in vitro libraries (see transcriptome analysis section below) together with ESTs from Vi1 grown in vitro and in planta (Vi1-infected susceptible apple leaves) [46] were used to train AUGUSTUS [23, 153] to build a species model file (meta parameter file) for *Venturia*. Hybrid (evidence-based) gene prediction was performed for Vi1 using this model together with the Vi1 transcript sequences and ESTs as hints in AUGUSTUS. Based on the trained species model, ab initio gene predictions were carried out on the repeat-masked genomes for all the five *Venturia* isolates, thus there were two sets of gene predictions for the Vi1 genome.

#### Transcriptome analysis

RNA-Seq data QC was done using FASTX-Toolkit v0.0.13 [142]. Reads were trimmed to 64 nucleotides based on the QC reports (using a median phred score >20 as threshold). For genome-guided transcriptome assembly, trimmed RNA-Seq reads from Vi1 in vitro and two or seven dpi (in planta) were mapped to the reference genome of Vi1 using tophat-1.4.1 [154] and further assembled with cufflinks-1.3.0 [155]. De novo transcriptome assembly was carried out using Inchworm (now evolved to Trinity) [156, 157], and Oases [158] to aid gene prediction (see above). To measure transcriptional support for genes FPKM values were calculated within samples enabling gross ranking of transcriptional activity, with an arbitrary threshold of >1 for evidence of transcription. Differential gene expression tests were carried out between in vitro and in planta samples at different inoculation time points, using rsem-1.2.4 [159] to first map the reads, followed by edgeR v3.2.4 [160] in Bioconductor version 2.12 [161]. Highly differentially expressed genes, with a false discovery rate <0.05 and log_2_ fold change (LogFC) not smaller than two, were used for prioritisation of genes for analysis.

#### Identification of the predicted secretomes

Genes encoding putatively secreted proteins were identified in each *Venturia* genome and gene catalogues from *P. nodorum*, *P. graminis* f. sp *tritici* and *C. fulvum* downloaded via the Mycocosm portal at the JGI, using a custom software pipeline (Additional file 6). Both the hybrid (based on trancriptome resources used to build the trained species model) and ab initio (based on the trained species model) predictions for Vi1 were used in the secretome discovery pipeline to avoid excluding genes predicted in only one approach; gene predictions in either set that co-localised at the same locus (with a greater than 90% identity and e value of <1e-10) were considered a single prediction. The SignalP 4.0 [162] and the SecretomeP [163] servers were used initially to screen for predicted proteins with a signal peptide or those secreted via a non-classical pathway, respectively. The TMHMM server was used to screen for predicted proteins without a predicted transmembrane domain [164]. Only those predicted proteins, either lacking a transmembrane domain or with a single transmembrane domain with at least 10 aa in the first 60 aa, indicating a probable correspondence with secretion signal, were considered for further analysis. The TargetP server [165] was used to predict cellular location, with those predicted to be extracellular retained. ProtComp v 9.0 [166] and WoLF PSORT servers (with an extracellular score threshold of >17) [167] were used to further analyse the putative location of these predicted proteins. Proteins predicted to be secreted by either method were included to avoid the omission of false negatives.

#### Annotation of the predicted secretomes

Putative functions of the secretome proteins were initially assigned following gene ontology analysis through an in-house annotation pipeline (BioView; Ross Crowhurst, Marcus Davy and Cecilia Deng, unpublished) that scans public databases including NCBI RefSeq [168], InterPro [169], UniRef [170], ExPASy UniProtKB/Swiss-Prot [171] and ExPASy Prosite [172] and subsequent manual curation on the basis of similarity searches (BLASTp with a similarity threshold of 1e-10) against the NCBI nr database [29]. In addition, identification of the complement of putative carbohydrate-active enzymes (CAZymes) was achieved using the CAT server [27]. Default parameters were used for CAZyme identification, apart from the -threshold which was set to 1e-05.

*Venturia* genomes were also interrogated (using a combination of automated annotations, BLASTp and tBLASTn, with a similarity threshold of 1e-10), with sequences listed in PHI-base [35] and other effectors (e.g. *V. dahliae* Ave1, GenBank: AFB18188.1; *L. maculans*, AvrLm6 GenBank: CAJ90695.1; *C. fulvum* Ecp6 GenBank: ACF19427.1), as well as sequences annotated as ‘hydrophobin’ and containing the ascomycete-specific hydrophobin motif IPR010636 or conserved eight cysteine pattern, from *C. fulvum* and *D. septosporum* (as referred to in [69]), *P. nodorum* (JGI protein ID 9201, SNOG_03122.3) and *L. maculans* (JGI protein ID 775, Lema_T007750.1). Conserved domains were identified using Pfamscan [36–38].

Orthologous protein clusters were identified using OrthoMCL version 2.0.3 [30] together with the Markov clustering algorithm mcl [173] to cluster all-vs-all BLASTp (e value <1e-10) results across all isolates.

#### Small secreted proteins (SSPs)

Small secreted proteins (SSPs) were defined as proteins that were present in the secretome dataset with a length of ≤500 aa (following removal of the secretion signal if identified by SignalP). Enzymes, such as CAZymes, were not included in the SSP set. A more stringent length parameter of 200 aa was also used to identify a more discrete set of candidate effectors. Those SSPs with two or more cysteine residues in the predicted mature protein were also identified. SSPs were screened for repeat content using RADAR [174–176]. The presence of pro-peptide cleavage sites was analysed using ProP 1.0 [177], with those sites with a score ≥0.5 recorded as positive. InterProScan 5 [174, 178] was used to analyse conserved domains in the SSPs.

In addition to the OrthoMCL analysis, the SSPs (≤200 aa in length) were analysed further using Spectral Clustering SCPS 0.9.8 [57] to detect possible clusters of SSPs comprising members with lower sequence similarity than those clustered using the more stringent thresholds of OrthoMCL. The parameters (default) used were those that resulted in the AvrLm6-like proteins forming a cluster with the same members as when clustered manually [45]. The clusters were manually inspected and those members with a similar cysteine pattern retained. In addition, since those SSPs less than 200 aa in length were over-represented in the Vi1 secretome, members of the clusters from Vi1 were used to detect possible gene predictions that were not called automatically by searching each of the *Venturia* genomes using genBlastG 1.39 [179], then manually curating novel gene models followed by family assignment based on cysteine patterns and construction of multiple sequence alignments using T-Coffee 11.00.8cbe486 [180, 181]. Sequence logos were constructed using the TeX package, TeXshade [182].

Proteins similar to selected SSPs in related Dothideomycete genomes (Additional file 15) were identified either by BLASTp for singletons or HMMER 3.1b2 [183] for families (thresholds 1e-10), and clustering data pertaining to the predicted Dothideomycete orthologues were obtained from the JGI. Dothideomycete genomes were selected for comparison based on phylogenetic spread and diversity of lifestyle, including extremophile saprobes, biotrophs, hemibiotrophs and necrotrophs.

#### Gene density and proximity to transposable elements (or transposable element remnants) in the Vi1 genome

Intergenic distances were calculated for the predicted genes of Vi1. Gene density across the Vi1 genome was approximated from 5′ and 3′ flanking intergenic lengths. Intergenic lengths were binned according to log (length) and plotted as a 2-dimensional heatmap in R [184] using a method adapted from Saunders and associates [185]. The average distances were also calculated; however, genes lacking an immediate neighbour at either the 5′ or 3′ flank, such as those at the ends of genomic scaffolds, were excluded from this analysis. The *V*IS set, plus *AvrLm6-* and *Ave1*-like genes were compared with 440 core eukaryotic genes [25].

The repeat prediction suite REPET 2.2 [186] was used for the detection and annotation of TEs (repeat sequences) for the whole genome sequence of Vi1. A custom Python script was used to calculate the 5′ and 3′ flanking distances between gene annotations and TE genomic features that were identified in the REPET analysis. Flanking distances between genes and repeats were binned according to log (length) and plotted as a 2-dimensional heatmap in R [184] using a method adapted from Saunders and associates [185]. The *V*IS and core eukaryotic gene gene sets were plotted for comparison. Student’s *t*-Tests were conducted to determine whether any differences were statistically significant.

## Additional files

Additional file 1: Statistics for the whole genome assemblies of isolates of *Venturia inaequalis* and *V. pirina*. (XLSX 11 kb)

Additional file 2: Repeats in *Venturia inaequalis* and *V. pirina* genome assemblies. (XLSX 10 kb)

Additional file 3: Analysis of gene models from the *Venturia inaequalis* and *V. pirina* genomes. (XLSX 10 kb)

Additional file 4: Core Eukaryotic Gene Model Analysis (CEGMA) of the *Venturia inaequalis* and *V. pirina* genomes. The 248 most highly-conserved core eukaryotic genes were used in the CEGMA to assess genome completeness. (XLSX 10 kb)

Additional file 5: Benchmarking Universal Single-Copy Orthologs (BUSCO_v1.1b) analysis of the *Venturia inaequalis* and *V. pirina* genomes. BUSCO analysis was used to assess completeness of the *Venturia* genome assemblies. (XLSX 10 kb)

Additional file 6: Pipeline of programmes used to identify the in silico-predicted secretome of each isolate. (DOCX 147 kb)

Additional file 7: The predicted secretomes and small, secreted proteins (SSPs) encoded by the genomes of *Venturia* and representative related pathogens. The genomes for *Parastagonospora nodorum*, *Cladosporium fulvum* and *Puccinia graminis* f. sp. *tritici* were downloaded via the Mycocosm portal at the Joint Genome Institute (JGI; see details in Additional file 15). These pathogens were representatives of necrotrophic, facultative and obligate biotrophic fungi, respectively. The pipeline outlined in Additional file 6 was employed to identify the secretome and SSPs in each of the genomes. (XLSX 11 kb)

Additional file 8: The predicted proteins belonging to OrthoMCL clusters present in the secretomes of five *Venturia* isolates. The coloured horizontal bars represent proportions of predicted proteins (scale at bottom of figure) that have been annotated according to the key at the top of the figure, that are present in the secretomes indicated by grey boxes to the left of the figure (white boxes indicate lack of similar proteins). S: indicates singleton proteins. Numbers in the boxes are numbers of proteins. The order of the categories in the key is the same as that in the horizontal bars. For example: the core secretome is represented by the top-most horizontal bar; there are similar proteins present in each of the secretomes (5 grey boxes), of which 15% (77 proteins) are classified as SSPs between 200 and 500 amino acids in length with 2 or more cysteines. (PPTX 56 kb)

Additional file 9: The CAZyme domains present in proteins encoded by the *Venturia* secretomes. CAZyme domains were identified using the CAZymes analysis toolkit (CAT) server. Domains: CBM: carbohydrate binding module; CE: carbohydrate esterase; GH: glycoside hydrolase; GT: glycosyltransferase; PL: polysaccharide lyase. The known substrates for the CAZyme domains were taken from Zhao et al. PCW: plant cell wall. FCW: fungal cell wall. CW: cell wall. ESR: energy storage and recovery. PG: protein glycosylation. dpi: days post inoculation. (XLSX 17 kb)

Additional file 10: Summary of distribution of similar proteins with CAZyme domains encoded by the *Venturia* secretomes. CAZyme domains were identified using the CAZymes analysis toolkit (CAT) server. Filled grey boxes in columns indicate presence of a similar protein or proteins in that secretome identified by OrthoMCL clustering. (XLSX 12 kb)

Additional file 11: Classification of proteases in the secretomes of the *Venturia* pathogens. Filled grey boxes in columns indicate presence of similar proteins in that secretome identified by OrthoMCL clustering: aspartate and serine (including those annotated as subtilases) proteases, metalloproteases and unclassified peptidases. Several of the proteases were similar (<e-10) to proteases in the Pathogen-Host Interactions database (PHI-base). (XLSX 15 kb)

Additional file 12: Summary of small, secreted proteins (SSPs) similar to proteins present in the Pathogen-Host Interactions database. Filled grey boxes in columns indicate presence of a similar protein(s) in that secretome identified by OrthoMCL clustering. (XLSX 12 kb)

Additional file 13: Small, secreted protein (SSP) family 07 members. Sequence logo of members of A. family 07 from Vi1 and B. family 07 from all isolates. (DOCX 221 kb)

Additional file 14: Details of the small (≤200 amino acids in length) secreted proteins in the *Venturia* infection secretome (*V*IS) gene set. (XLSX 22 kb)

Additional file 15: The genomes used in this study (non-*Venturia*). Sources of the genomes downloaded or accessed (with permission) from the Joint Genome Institute (JGI)/MycoCosm. (XLSX 14 kb)

Additional file 16: Details of the lineage-specific small, secreted proteins (SSPs) identified in *Venturia inaequalis* ViL and *V. pirina* secretomes. Only those proteins ≤200 amino acids in length are included. (XLSX 18 kb)

Additional file 17: Details of isolates of *Venturia inaequalis* and *V. pirina* used in this study. (XLSX 12 kb)

## Abbreviations

aa: Amino acid
AGRF: Australian Genome Research Facility
AWCGS: Allan Wilson Centre Genome Service
BUSCO: Benchmarking Universal Single-Copy Orthologs
CAT: CAZyme Analysis Toolkit
CAZyme: Carbohydrate Active Enzyme
CBD: Chitin-binding domain
CBM: Carbohydrate-binding motif
CE: Carbohydrate esterase
CEGMA: Core Eukaryotic Genes Mapping Approach
*Cin*: Cellophane-induced
dpi: Days post inoculation
FPKM: Fragments per kilobase of exon per million reads mapped
gDNA: Genomic DNA
GH: Glycoside hydrolase
GT: Glycosyl transferase
hpi: Hours post inoculation
HR: Hypersensitive response
HsbA: hydrophobic surface binding
JGI: Joint Genome Institute
LysM: Lysin motif
Mimp: Miniature impala transposon
MP: Mate-pair
NLP: Nep-1-like protein
PAMP: Pathogen-associated molecular pattern
PBSA: Poly(butylene succinate-co-adipate)
PDA: Potato dextrose agar
PE: Paired-end
PFR: Plant & Food Research Limited
PL: Polysaccharide lyase
PTI: PAMP-triggered immunity
QC: Quality check
R: Resistance
RIP: Repeat induced point mutation
SDW: Sterile distilled water
SSP: Small, secreted, non-enzymatic protein
TEs: Transposable elements
VICE: *V. inaequalis* candidate effector
*V*IS: *Venturia* Infection Secretome
VLSCI: Victorian Life Sciences Computation Initiative
