# Supplementary material for: Comparative analysis of the predicted secretomes of Rosaceae scab pathogens Venturia inaequalis and V. pirina reveals expanded effector families and putative determinants of host range
Source: BMC Genomics. 2017 May 2;18:339. doi: 10.1186/s12864-017-3699-1 (PMC5412055; doi:10.1186/s12864-017-3699-1)
Supplement: Supplementary file 6 — Pipeline of programmes used to identify the in silico-predicted secretome of each isolate. (DOCX 147 kb) [file 12864_2017_3699_MOESM6_ESM.docx]

**Additional file 6:**

**
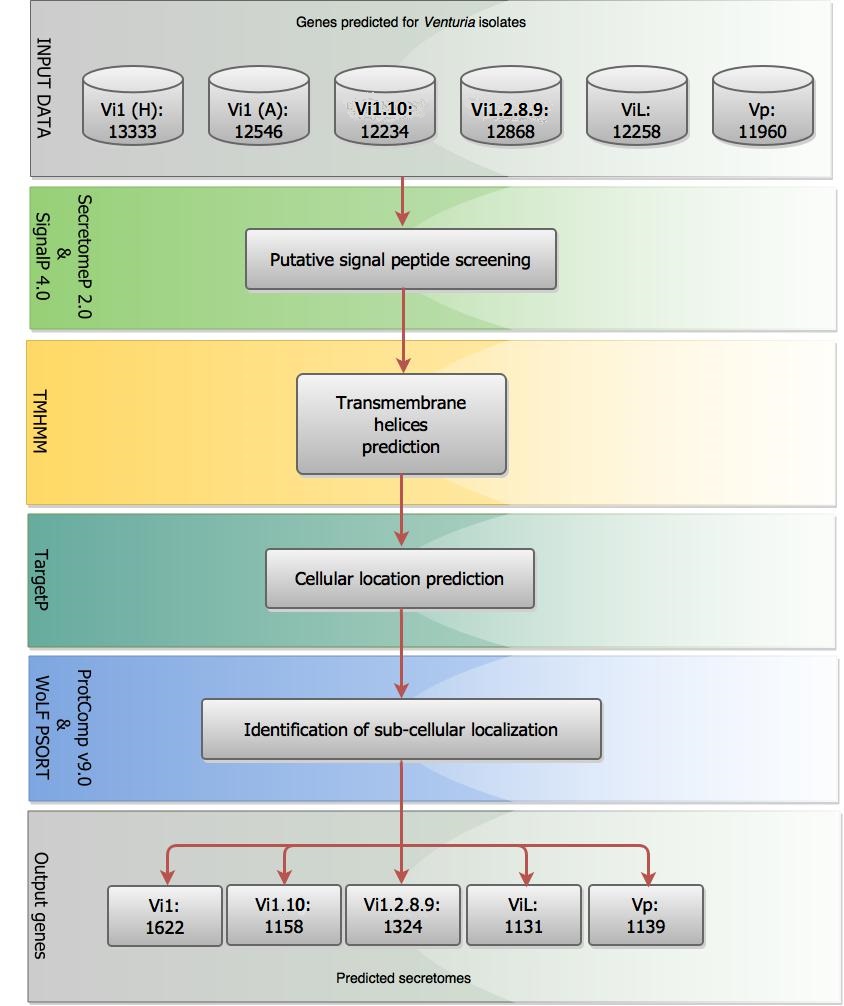
**

Both the hybrid, Vi1 (H), and ab initio, Vi1 (A), predictions for Vi1 were used in the secretome discovery pipeline to avoid excluding candidate effectors predicted in only one approach. Gene predictions that co-localised at the same locus were show [?A3B2 show $132#?]

considered a single prediction (with a greater than 90% identity and e value of <1e-10). The SignalP 4.0 [Petersen TN, Brunak S, von Heijne G, show [?A3B2 twb=.27w?]

show [?A3B2 tlsb=-.09pt?]

Nielsen H. SignalP 4.0: discriminating signal peptides from transmembrane regions. Nat Methods. 2011;8:785-6.] and the SecretomeP [Bendtsen JD, show [?A3B2 show $9#?]

Jensen LJ, Blom N, von Heijne G, Brunak S. Feature-based prediction of non-show [?A3B2 twb=.27w?]

show [?A3B2 tlsb=-.15pt?]

classical and leaderless protein secretion. Protein Eng Des Sel. 2004;17:349-56] servers were used initially to screen for predicted proteins with a signal show [?A3B2 show $132#?]

peptide or those secreted via a non-classical pathway, respectively. The TMHMM server was used to screen for predicted proteins without a predicted transmembrane domain [The TMHMM server. http://www.cbs.dtu.dk/services/TMHMM/. Accessed: Vi1 07.04.2011; Vi1.10 03.04.2013; ViL and Vi1.2.8.9 24.04.2013; Parastagonospora nodorum 29.10.2015; Cladosporium fulvum 11.09.2015; Puccinia graminis f.sp. tritici 10.09.2015]. Only those predicted proteins, either lacking a transmembrane domain or with a single transmembrane domain with at least ten amino acids in the first 60 amino acids, indicating a probable show [?A3B2 show $132#?]

correspondence with secretion signal, were considered for further analysis. The TargetP server [Emanuelsson O, Nielsen H, Brunak S, von Heijne G. show [?A3B2 show $9#?]

Predicting subcellular localization of proteins based on their N-terminal amino acid sequence. J Mol Biol. 2000;300:1005-16] was used to predict cellular show [?A3B2 show $9#?]

location, with those predicted to be extracellular retained. ProtComp v 9.0 [http://www.softberry.com/berry.phtml?topic=protcomppl&group=programs&subgroup=proloc. Accessed: Vi1 10.04.2011; Vi1.10 07.04.2013; ViL and Vi1.2.8.9 28.04.2013; Parastagonospora nodorum 04.11.2015; Cladosporium fulvum 16.09.2015; Puccinia graminis f.sp. tritici 14.09.2015] and WoLF PSORT servers (with an show [?A3B2 twb=.4w?]

show [?A3B2 tlsb=.09pt?]

extracellular score threshold of >17) [Horton P, Park K-J, Obayashi T, Fujita N, Harada H, Adams-Collier CJ, et al. WoLF PSORT: protein localization predictor. Nucleic Acids Res. 2007;35 Web Server issue:W585-7] were used to further analyse the putative location of these predicted proteins.
