# Supplementary material for: Comparative analysis of the predicted secretomes of Rosaceae scab pathogens Venturia inaequalis and V. pirina reveals expanded effector families and putative determinants of host range
Source: BMC Genomics. 2017 May 2;18:339. doi: 10.1186/s12864-017-3699-1 (PMC5412055; doi:10.1186/s12864-017-3699-1)
Supplement: Supplementary file 8 — The predicted proteins belonging to OrthoMCL clusters present in the secretomes of five Venturia isolates. The coloured horizontal bars represent proportions of predicted proteins (scale at bottom of figure) that have been annotated according to the key at the top of the figure, that are present in the secretomes indicated by grey boxes to the left of the figure (white boxes indicate lack of similar proteins). S: indicates singleton proteins. Numbers in the boxes are numbers of proteins. The order of the categories in the key is the same as that in the horizontal bars. For example: the core secretome is represented by the top-most horizontal bar; there are similar proteins present in each of the secretomes (5 grey boxes), of which 15% (77 proteins) are classified as SSPs between 200 and 500 amino acids in length with 2 or more cysteines. (PPTX 56 kb) [file 12864_2017_3699_MOESM8_ESM.pptx]

## Slide 1
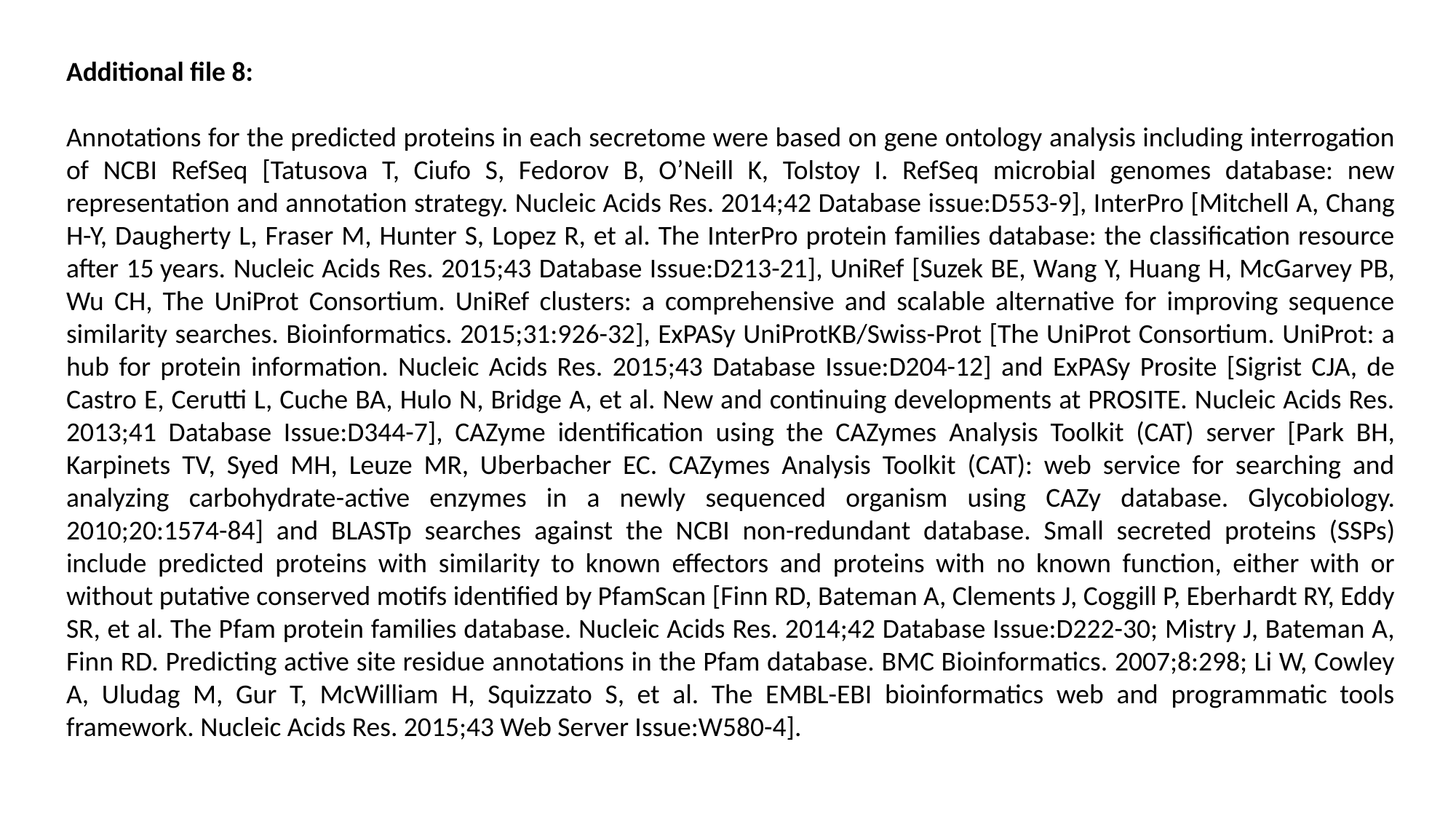

Additional file 8:
Annotations for the predicted proteins in each secretome were based on gene ontology analysis including interrogation of NCBI RefSeq [Tatusova T, Ciufo S, Fedorov B, O’Neill K, Tolstoy I. RefSeq microbial genomes database: new representation and annotation strategy. Nucleic Acids Res. 2014;42 Database issue:D553-9], InterPro [Mitchell A, Chang H-Y, Daugherty L, Fraser M, Hunter S, Lopez R, et al. The InterPro protein families database: the classification resource after 15 years. Nucleic Acids Res. 2015;43 Database Issue:D213-21], UniRef [Suzek BE, Wang Y, Huang H, McGarvey PB, Wu CH, The UniProt Consortium. UniRef clusters: a comprehensive and scalable alternative for improving sequence similarity searches. Bioinformatics. 2015;31:926-32], ExPASy UniProtKB/Swiss-Prot [The UniProt Consortium. UniProt: a hub for protein information. Nucleic Acids Res. 2015;43 Database Issue:D204-12] and ExPASy Prosite [Sigrist CJA, de Castro E, Cerutti L, Cuche BA, Hulo N, Bridge A, et al. New and continuing developments at PROSITE. Nucleic Acids Res. 2013;41 Database Issue:D344-7], CAZyme identification using the CAZymes Analysis Toolkit (CAT) server [Park BH, Karpinets TV, Syed MH, Leuze MR, Uberbacher EC. CAZymes Analysis Toolkit (CAT): web service for searching and analyzing carbohydrate-active enzymes in a newly sequenced organism using CAZy database. Glycobiology. 2010;20:1574-84] and BLASTp searches against the NCBI non-redundant database. Small secreted proteins (SSPs) include predicted proteins with similarity to known effectors and proteins with no known function, either with or without putative conserved motifs identified by PfamScan [Finn RD, Bateman A, Clements J, Coggill P, Eberhardt RY, Eddy SR, et al. The Pfam protein families database. Nucleic Acids Res. 2014;42 Database Issue:D222-30; Mistry J, Bateman A, Finn RD. Predicting active site residue annotations in the Pfam database. BMC Bioinformatics. 2007;8:298; Li W, Cowley A, Uludag M, Gur T, McWilliam H, Squizzato S, et al. The EMBL-EBI bioinformatics web and programmatic tools framework. Nucleic Acids Res. 2015;43 Web Server Issue:W580-4].

## Slide 2
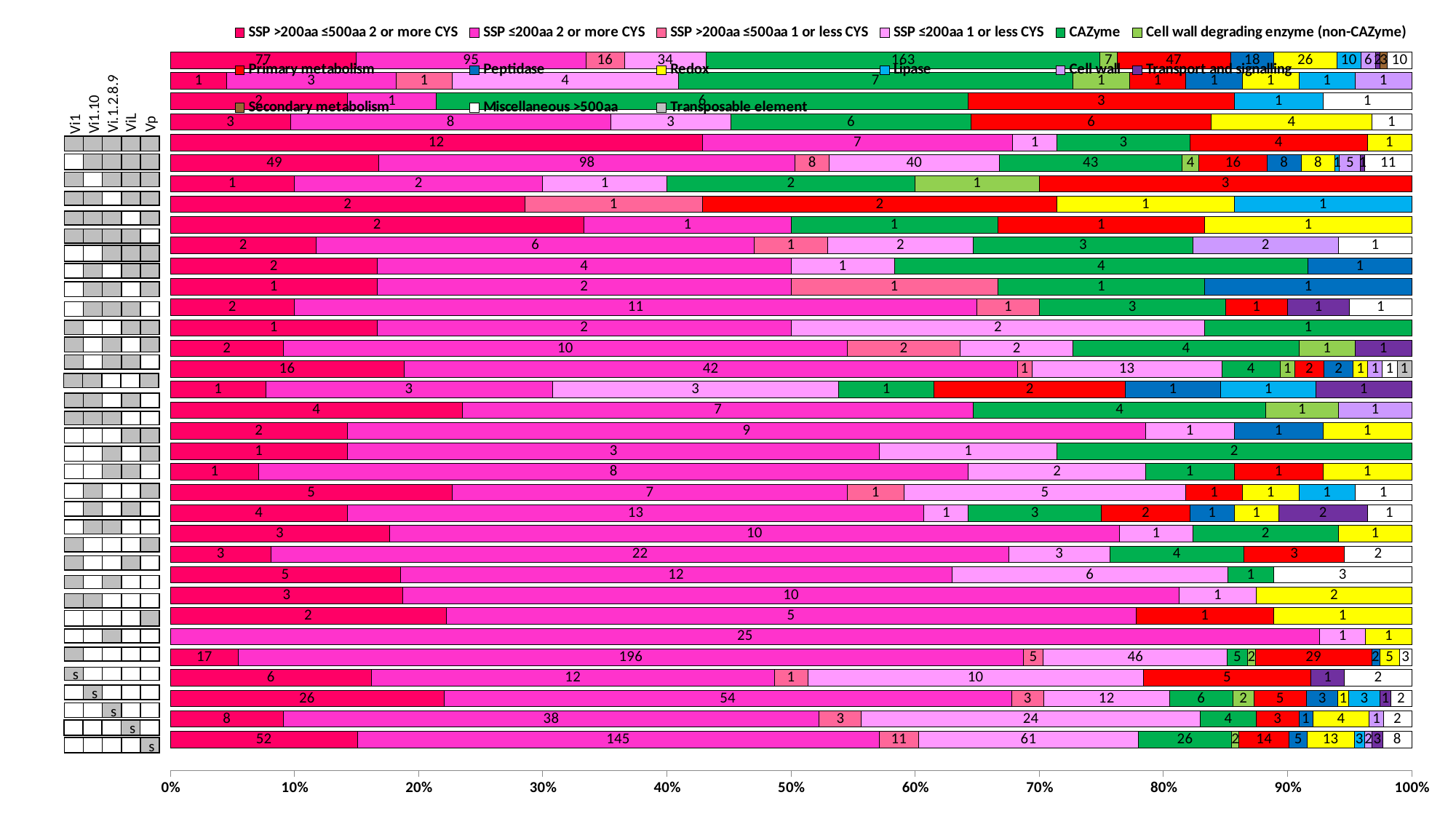

### Chart
| Category | SSP >200aa ≤500aa 2 or more CYS | SSP ≤200aa 2 or more CYS | SSP >200aa ≤500aa 1 or less CYS | SSP ≤200aa 1 or less CYS | CAZyme | Cell wall degrading enzyme (non-CAZyme) | Primary metabolism | Peptidase | Redox | Lipase | Cell wall | Transport and signalling | Secondary metabolism | Miscellaneous >500aa | Transposable element |
|---|---|---|---|---|---|---|---|---|---|---|---|---|---|---|---|Vi.1.2.8.9
Vi1.10
ViL
Vp
Vi1
s
s
s
s
s
