# Supplementary material for: Comparative analysis of the predicted secretomes of Rosaceae scab pathogens Venturia inaequalis and V. pirina reveals expanded effector families and putative determinants of host range
Source: BMC Genomics. 2017 May 2;18:339. doi: 10.1186/s12864-017-3699-1 (PMC5412055; doi:10.1186/s12864-017-3699-1)

**Additional file 13:**

show [?A3B2 tlb=9.5pt?]

Small, secreted protein (SSP) family 07 members. Sequence logo of members of A. family 07 from Vi1 and B. family 07 from all isolates. Sequence logos were constructed using the TeX package, TeXshade [Beitz E. TeXshade: shading and labeling of multiple sequence alignments using LaTeX2e. Bioinformatics. 2000;16:135-9]. Each position is displayed as a stack of residue symbols whose heights represent their proportion of the information content.


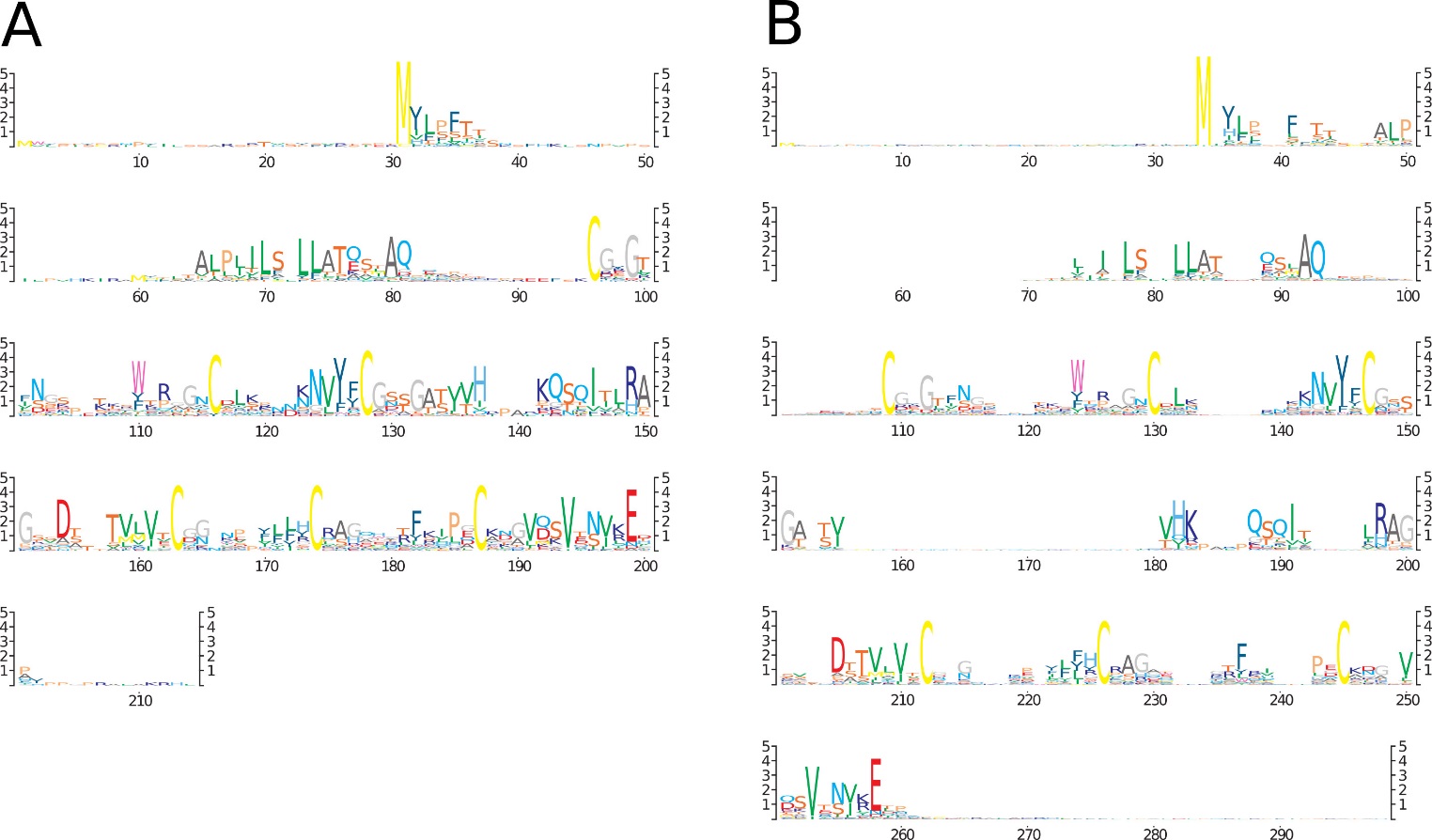

Supplement: Supplementary file 13 — Small, secreted protein (SSP) family 07 members. Sequence logo of members of A. family 07 from Vi1 and B. family 07 from all isolates. (DOCX 221 kb) [file 12864_2017_3699_MOESM13_ESM.docx]
